# Light-triggered multifunctional nanoplatform for efficient cancer photo-immunotherapy

**DOI:** 10.1186/s12951-022-01388-8

**Published:** 2022-04-07

**Authors:** Juan Yue, Qian Mei, Panyong Wang, Peng Miao, Wen-Fei Dong, Li Li

**Affiliations:** 1 School of Biomedical Engineering (Suzhou), Division of Life Sciences and Medicine, University of Science and Technology of China, Hefei, 230026 China; 2 CAS Key Laboratory of Biomedical Diagnostics, Suzhou Institute of Biomedical Engineering and Technology, Chinese Academy of Science (CAS), Suzhou, 215163 China

**Keywords:** Photo-immunotherapy, Hexahedron zinc porphyrin mesoporous nanoparticles, PD-L1 checkpoint blockade, Dendritic cell, Immune response

## Abstract

Cancer immunotherapy is limited by the immune escape of tumor cells and adverse effects. Photo-immunotherapy, the combination of immunotherapy and phototherapy (such as photodynamic therapy (PDT) and photothermal therapy (PTT)), can improve the effectiveness of immunotherapy in cancer treatment. Here, we first explored mesoporous hexagonal core–shell zinc porphyrin-silica nanoparticles (MPSNs), which are composed of a zinc porphyrin core and a mesoporous silica shell, and exhibit high laser-triggered photodynamic and photothermal activity, as well as outstanding drug loading capacity. In other words, MPSNs can be used not only as excellent photosensitizers for photo-immunotherapy, but also as an ideal drug carrier to achieve more efficient synergy. After loading with R837 (imiquimod, a toll-like receptor-7 agonist), MPSNs@R837 will elicit high-efficiency immunogenic cell death via PDT and PTT, and promote dendritic cell maturation after the PH-responsive release of R837, thereby, inducing tumor-specific immune responses. When combined with a programmed death ligand-1 checkpoint blockade, the photo-immunotherapy system markedly restrains primary tumors and metastatic tumors with negligible systemic toxicity. Therefore, the therapeutic strategy of integrating PTT, PDT and checkpoint blockade, shows great potential for suppressing cancer metastasis.

**Graphical Abstract:**

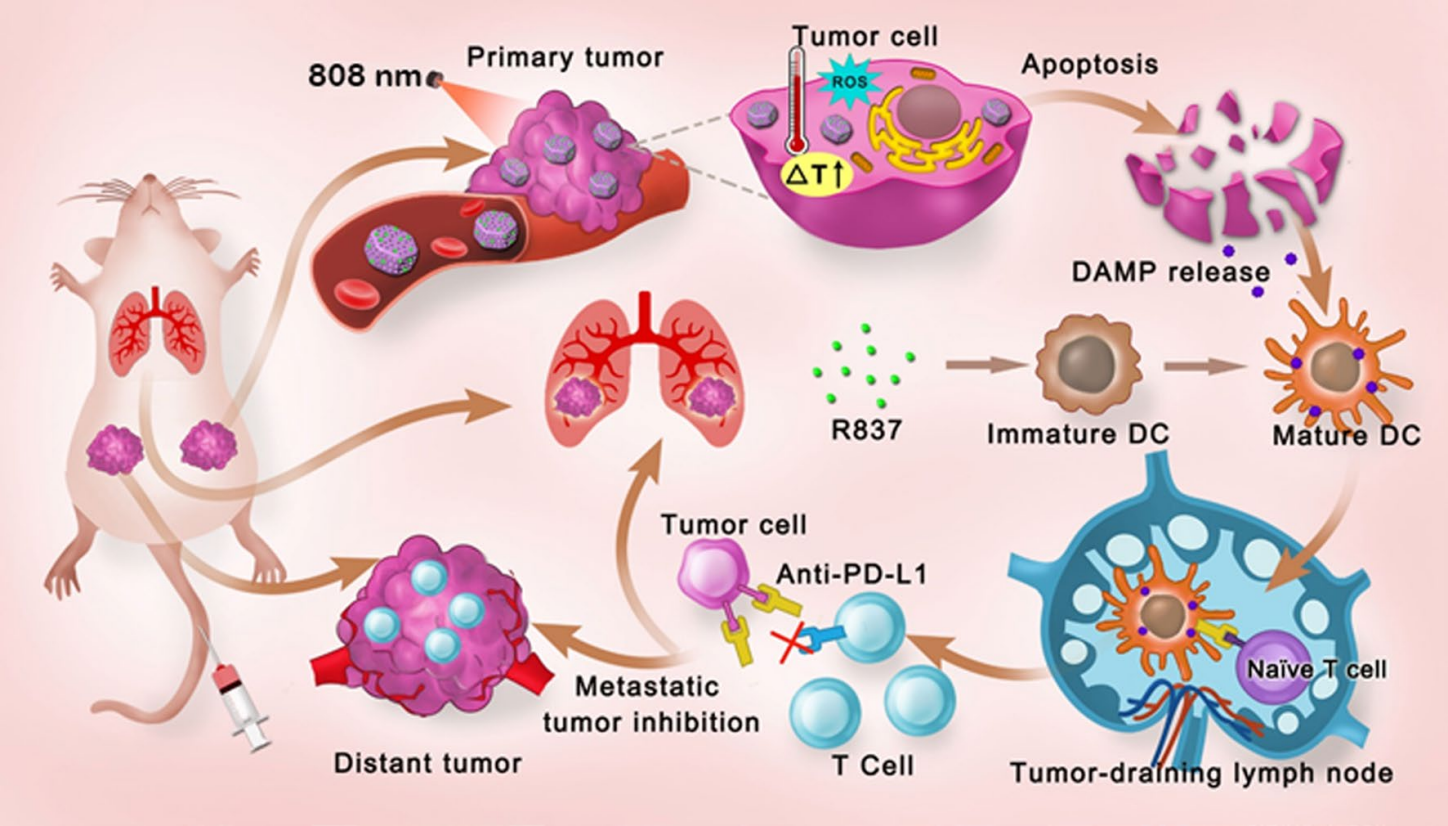

**Supplementary Information:**

The online version contains supplementary material available at 10.1186/s12951-022-01388-8.

## Introduction

Cancer immunotherapy serves as a clinical modality against tumor growth and metastasis by stimulating host immunological responses, which has achieved great progress in the field over the past few years [1–4]. However, immunotherapy still faces challenges such as immune-related adverse effects and low therapeutic responses [5–8]. Therefore, the integration of immunotherapy with various therapeutic modalities has attracted substantial attention [9–11] .

Phototherapy, including photodynamic therapy (PDT) and photothermal therapy (PTT), is one of the least invasive treatments, especially compared to chemotherapy [12–17]. PDT and PTT-induced immunogenic cell death (ICD) is a particular form of cell death [18, 19], that is characterized by the release of tumor-associated antigens and damage-associated molecular patterns [20], such as the translocation of calreticulin (CRT) and pro-inflammatory cytokines [21, 22], stimulating an immune response [23]. Although phototherapy can inhibit the growth of primary tumors [24, 25], the ability of stimulating immune response is rather weak [26].

Photo-immunotherapy-the combination of phototherapy and immunotherapy-can effectively enhance treatment effectiveness compared with a single treatment modality [27–30]. In recent years, increasing studies have reported that a variety of nanosystems as photosensitizers are used in photo-immunotherapy, such as noble metal nanoparticles [31], organic nanocarriers [32, 33], upconversion nanoparticles [34], and inorganic nanoparticles [35], etc. Owing to their unique optical properties, these nanoparticles can be used as excellent laser-triggered mediators [36]. Furthermore, by combining immune modalities, the emerging photo-immunotherapy approach partially suppresses the growth of primary tumors, and inhibits tumor recurrence and metastasis by activating the immune system [37]. However, most of these nanosystems use only a single PDT or PTT model to induce a relatively limited immune response [38]. Moreover, it is easy to ignore that antigen-presenting cells, such as dendritic cells (DCs), are immature due to the tumor immunosuppressive microenvironment, and their function in initiating an immune response is markedly hindered [39, 40]. Thus, it is often necessary to induce DC maturation using toll-like receptor (TLR) agonists [41, 42]. Although several studies have reported recent progress, the construction of a simple but multifunctional photo-immune system is still in its infancy due to the relatively limited function of known nanosystems [43–45]. To date, there is no relevant report on PDT combined with PTT, further integrating TLR to stimulate the immune response. Therefore, the development of a multifunctional and safe photo-immunotherapy system for efficient tumor treatment is urgently needed.

**Scheme 1:**
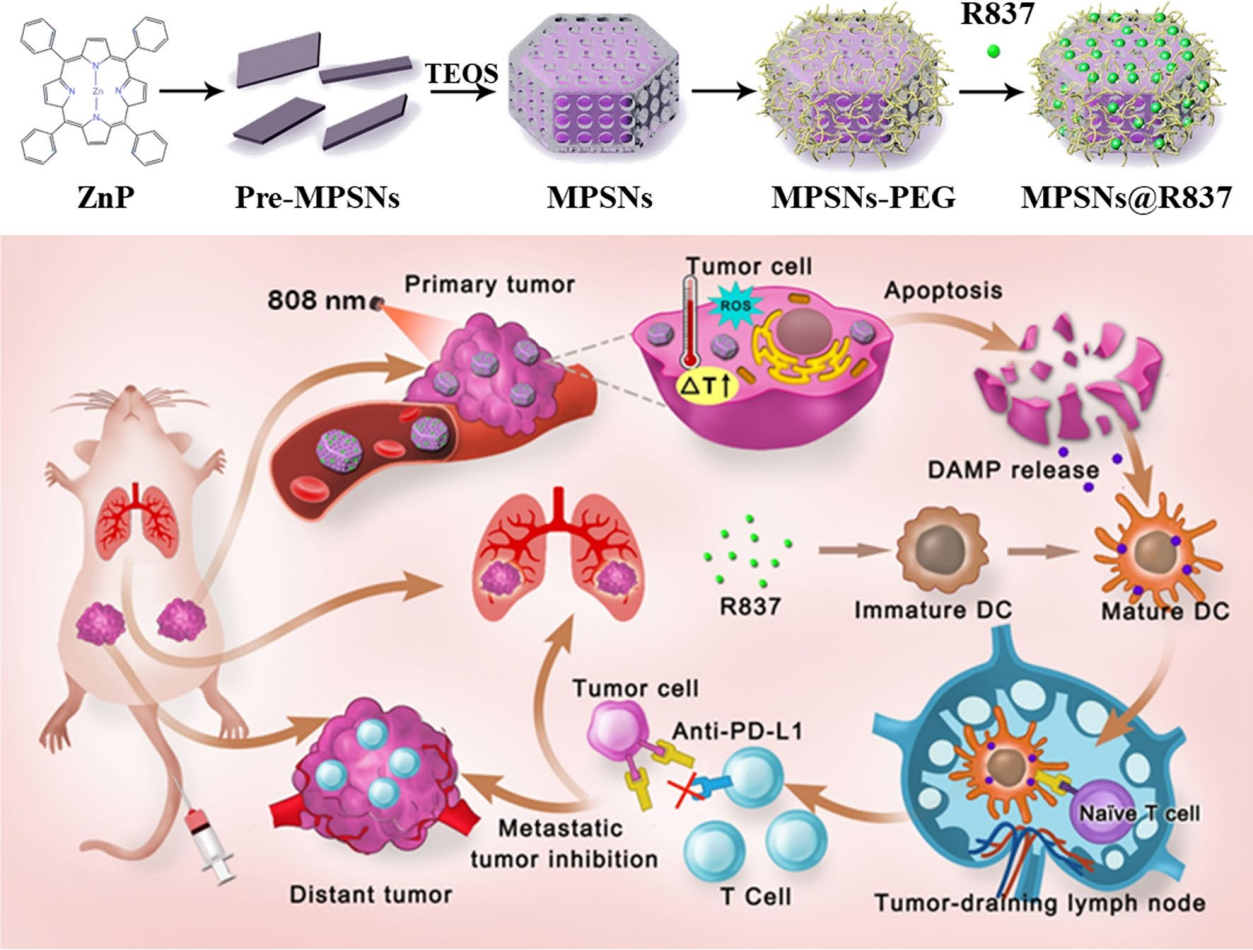
A schematic of the synthetic procedure for core–shell zinc porphyrin nanoplatform (MPSNs@R837) and the schematic illustration of MPSNs@R837 for combined phototherapy (PDT and PTT) and checkpoint blockade to enhance synergistic antitumor immunity

In this study, we first developed a multifunctional nanoplatform based on mesoporous hexagonal core–shell zinc porphyrin-silica nanoparticles (MPSNs) loaded with R837 (a toll-like receptor-7 agonist), which could be used to integrate PDT, PTT, and tumor-specific immunotherapy for breast cancer. MPSNs with ZnP as the core and a mesoporous silica framework as the shell can effectively generate singlet oxygen and convert photons to heat energy under only one light source, making the operation easier and safer. Meanwhile, the excellent mesoporous structure of the silica shell can facilitate efficient R837 loading. Taken together, MPSNs are not only excellent photosensitizers, but also efficient drug carriers. The immune adjuvant R837, functionalized together with tumor-associated antigens derived from primary tumors, is used to promote DC maturation, eliciting a strong immune response. Furthermore, combined with a programmed-death ligand-1 (PD-L1) checkpoint blockade, the novel nanoplatform showed more conspicuous anti-metastatic performance in 4T1 tumor-bearing mice. Therefore, the therapeutic strategy based on MPSNs@R837 not only eradicated primary tumors via phototherapy modalities (PDT and PTT), but also effectively inhibited distant metastasis due to the strong immune response triggered by the two-way mechanistic interaction (Scheme 1).

**Fig. 1:**
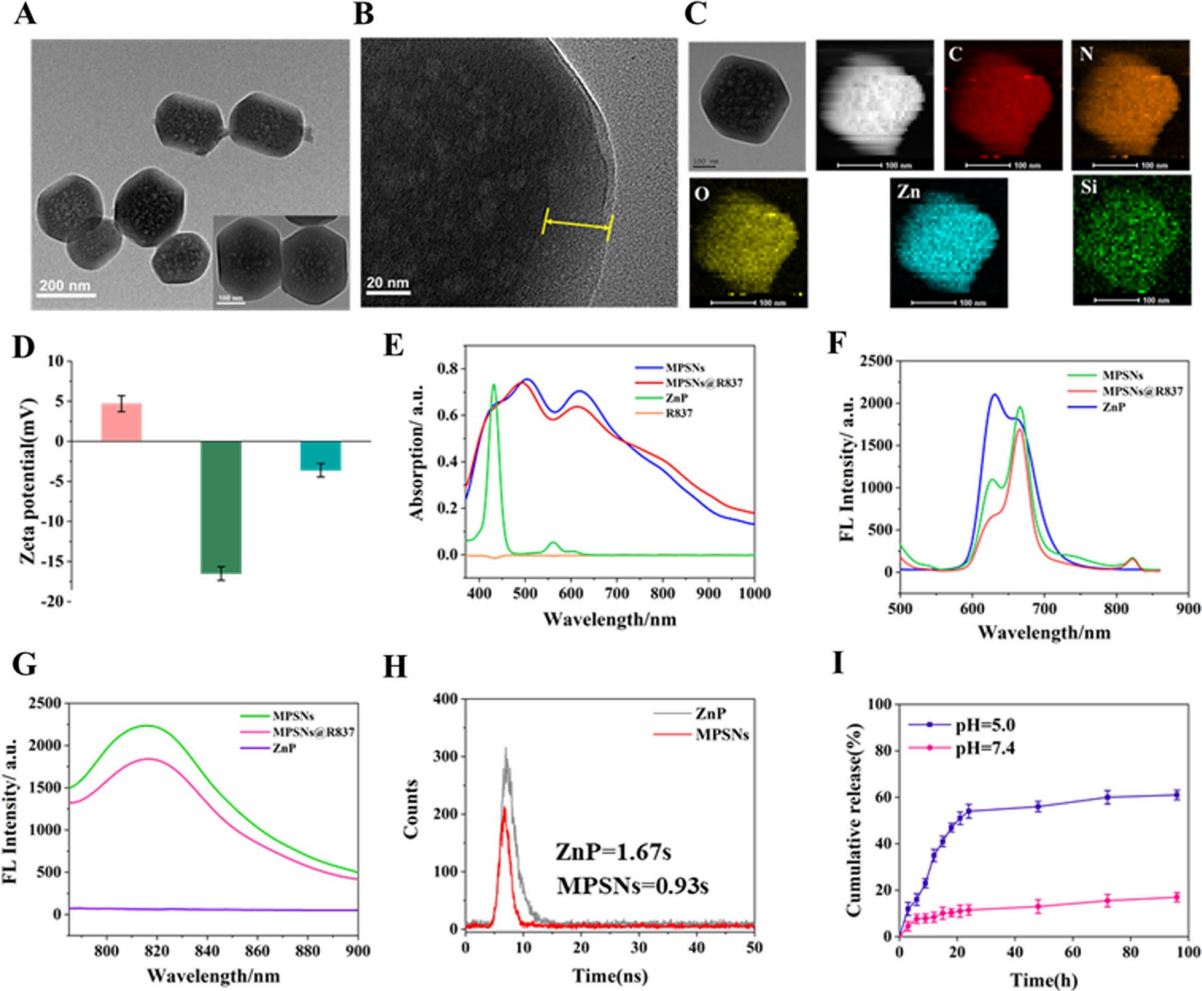
Characterization of MPSNs. **A** and **B** TEM images of MPSNs. **C** Elemental mapping of C, N, O, Zn, and Si of MPSNs. **D** Zeta potential. **E** UV/Vis spectra of MPSNs, MPSNs@R837, ZnP and free R837. **F** The fluorescence spectra of MPSNs, MPSNs@R837, and ZnP upon irradiation of 450 nm light, **G** upon irradiation of 780 nm light. **H** The fluorescence lifetime of ZnP and MPSNs in water. **I** R837 release from MPSNs@R837 at different pH values (pH 7.4 or 5.0)

A modified sol–gel method was used to synthesize the hexahedron-structure-like nanoplatform with self-assembled ZnP as the core and a mesoporous silica framework as the shell. In brief, ZnP was dissolved in an aqueous solution containing the surfactant cetyltrimethylammonium bromide (CTAB) and reacted for 24 h to form pre-MPSNs at room temperature during the first step. Subsequently, tetraethyl orthosilicate (TEOS) and a small amount of 3-aminopropyltriethoxysilane (APS) were slowly injected into the pre-MPSNs aqueous solution and stirred for 1 h at 40 ℃. TEM images of pre-MPSNs reveal fragmented structures in a 15-nm in diameter following the self-assembly of ZnP monomers (Additional file 1: Fig S1A, B), which further assembled into MPSNs cores. These images of the MPSNs clearly show a hexagonal core–shell morphology, with a total size of approximately 220 nm, and a shell thickness of approximately 30 nm with small pores (Fig. 1A, B; Additional file 1: Fig S1B). The MPSNs were dispersed in PBS without any aggregation over 7 days, demonstrating their excellent stability in aqueous solution (Additional file 1: Fig S1C). The elemental mapping images confirm the distributions of the major elements (C, N, O, Zn and Si) (Fig. 1C), which are consistent with the EDS results (Additional file 1: Fig S1D). Fig S1E and S1F illustrate that the Brunauer-Emmet-Teller surface area, total pore volume, and average pore size of the MPSNs are 636.48 m^2^ g^−1^, 1.90 cm^3^ g^−1^, and 12.06 nm, respectively. The zeta potentials of MPSNs, MPSNs-COOH and MPSNs@R837 are 5.6 mV, − 16.5 mV, and − 4.8 mV, respectively, indicating that the processes of FA-PEG-COOH functionalization and R837 loading are successful (Fig. 1D).

Then, the UV absorption spectra were determined (Fig. 1E), which reveal the ZnP’s typical Soret band at 425 nm. Unexpectedly, except for the Soret band at 420 nm, both MPSNs and MPSNs@R837 show another two strong Q bands at 500 nm and 625 nm. The nanoparticles have obvious absorption at 400 nm-800 nm. In addition, negligible changes were found in terms of R837. Fluorescence emission spectra reveal that ZnP, MPSNs and MPSNs@R837 all have strong fluorescence emission following 450 nm excitation, with several emission peaks at 625 nm and 675 nm (Fig. 1F). Interestingly, under 780 nm irradiation, MPSNs and MPSNs@R837 exhibit a strong fluorescence emission at 825 nm, whereas little fluorescence was observed for ZnP, which is attributed to the aggregation-caused emission (Fig. 1G). The fluorescence quantum yield of ZnP was measured as 11.2%, and that of the MPSNs was 10.68%, whose fluorescence lifetimes are 1.67 s and 0.93 s, respectively (Fig. 1H). The decline of fluorescence quantum yield and lifetime demonstrates the formation of aggregates. Notably, the fluorescence intensity of MPSNs has no obvious change under different pH values. At the same time, the fluorescence intensity decreased less than 10%, and the particle size distribution remained uniform after 10 days, whether in PBS or FBS (Additional file 1: Fig S2). The above results proved the excellent stability of MPSNs. The loading efficiency of R837 is calculated as 21.1%. In addition, MPSNs@R837 exhibit rapid R837 release (~ 48.5% after 20 h) at pH 5.0, and ~ 58.6% is released over 30 h. Whereas, less than 20% is released at pH 7.4 (Fig. 1I).

The ROS-generation capability of MPSNs in aqueous solution after 808 nm laser irradiation (0.6 W/cm^2^) was estimated by the ROS sensitive green fluorescent probe, named singlet oxygen sensor green (SOSG), whose fluorescence enhancement can characterize the content of ROS. Compared with the water sample, the MPSNs and MPSNs@R837 exhibit a noticeable fluorescence enhancement of SOSG, which proves their efficient ROS generation ability, indicating the potential for PDT in cancer treatment (Additional file 1: Fig S3).

**Fig. 2:**
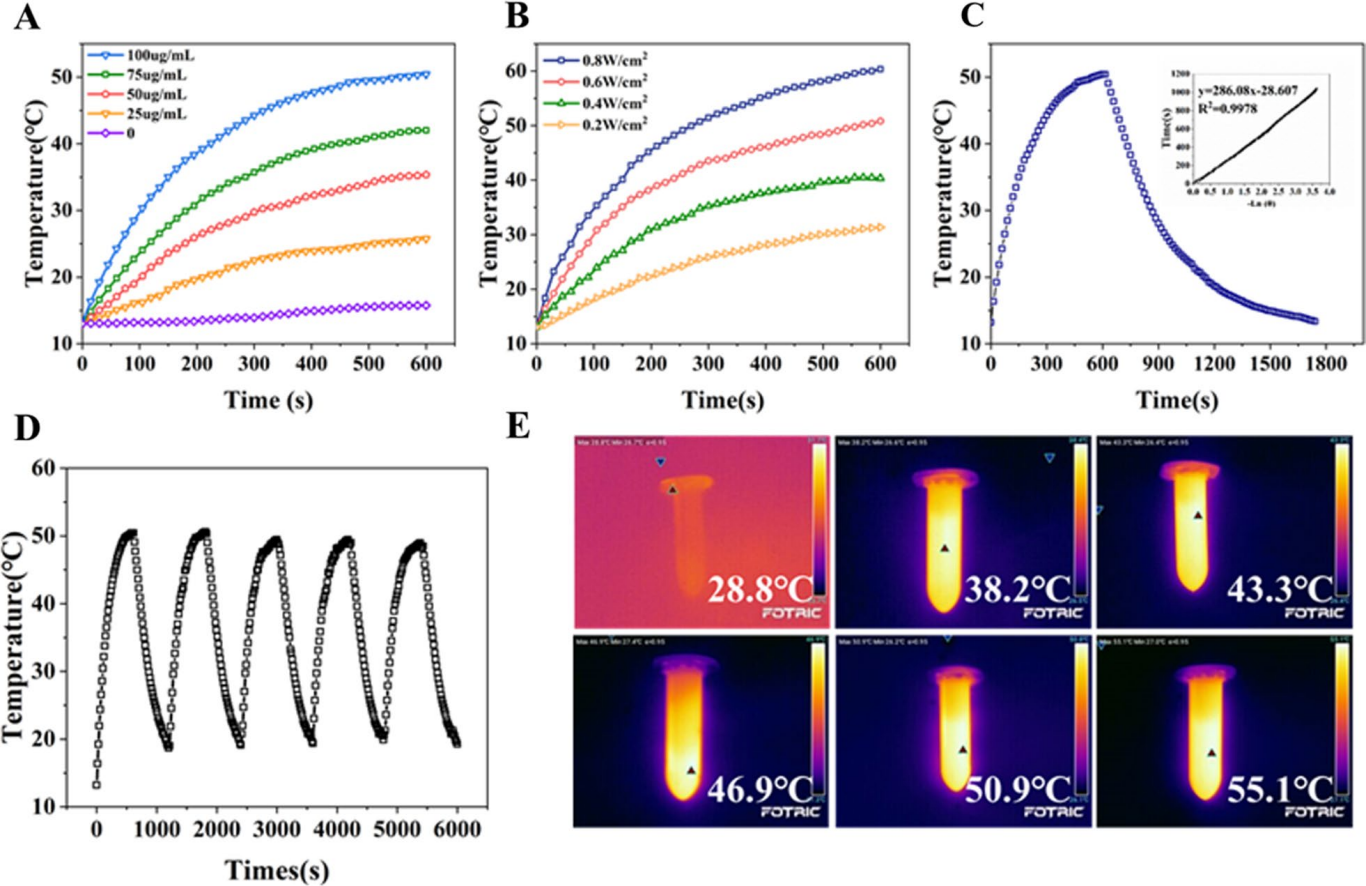
Photothermal heating curves of MPSNs aqueous solution (**A**) with different concentrations and (**B**) with different laser power densities. **C** Photothermal effect of the MPSNs solution (100 μg/mL) under irradiation of 808 nm laser (0.6 W/cm^2^) for 600 s min and left to cool down then, inset: Linear time data versus negative natural logarithm of the temperature driving force which is obtained from the cooling stage. **D** Temperature variations of the MPSNs under irradiation (808 nm, 0.6 W/cm^2)^ for five light on/off cycles (600 s of irradiation for each cycle). **E** Photothermal images of MPSNs in solution under laser irradiation

Next, the photothermal activity of MPSNs was explored. Figure 2A, B describe the temperature changes of MPSNs under different concentrations and laser intensities, which proves that the increase of temperature is concentration and power dependent. Meanwhile, compared to the negligible temperature change of water without MPSNs, the temperature of the MPSNs distinctly increased from 14.7 ℃ (the environmental temperature) to 50.2 ℃, demonstrating that the heat generation originated from the MPSNs. The photothermal conversion efficiency (η) of the MPSNs is determined by monitoring the temperature changes of the MPSNs between on and off of the laser irradiation. Figure 2C shows a plot between the cooling time after the laser off and the negative natural logarithm of the temperature change. The conversion efficiency is calculated as 43.8% according to a standard method. Surprisingly, MPSNs undergo no significant temperature changes even after five cycles of irradiation and cooling, which verifies their excellent photothermal stability (Fig. 2D). Thermal images show the significant temperature increases of MPSNs under laser irradiation compared with the irradiation of water alone (Fig. 2E).

**Fig. 3:**
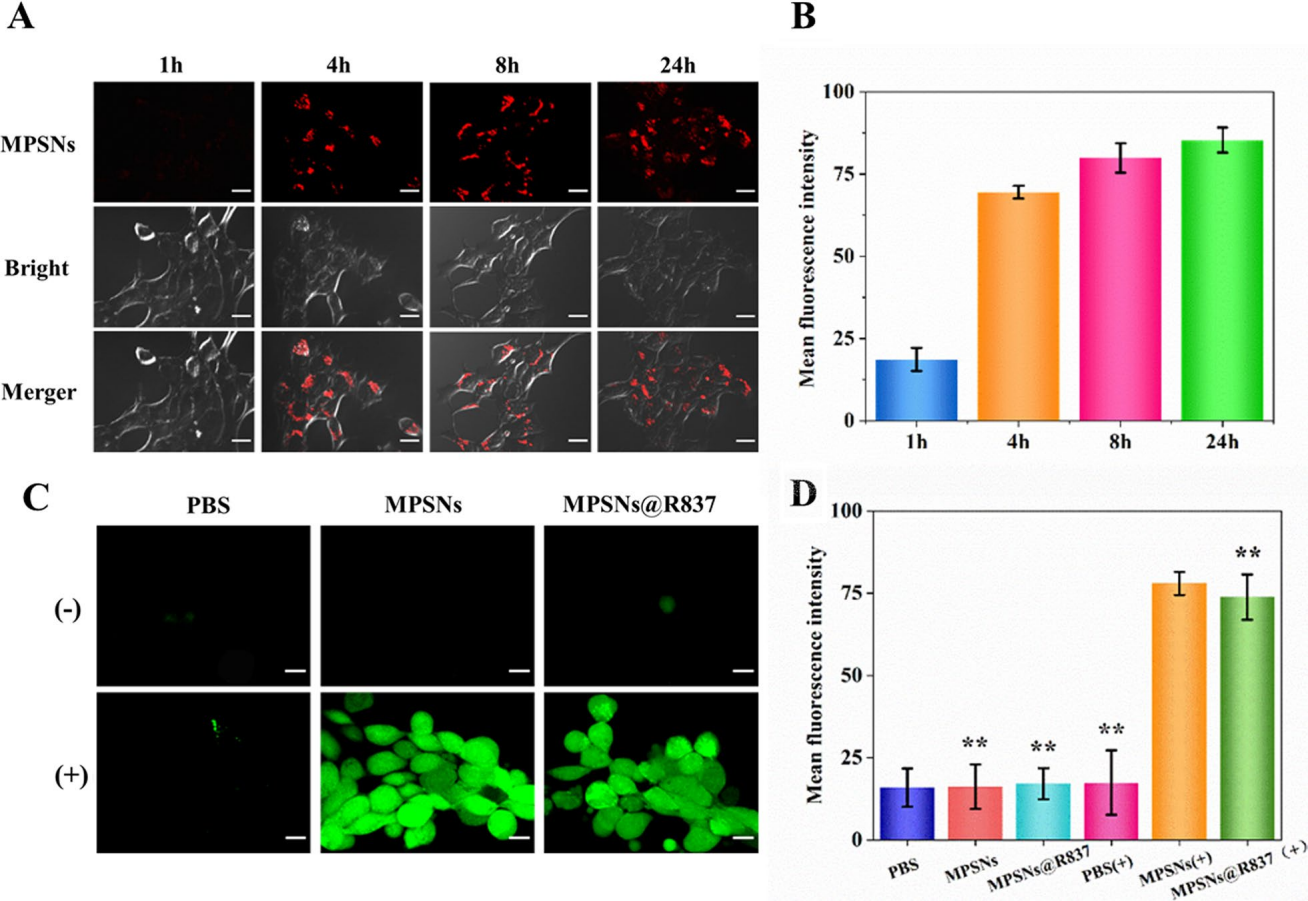
**A** CLSM images of 4T1 treated with MPSNs for different time (scale bar = 20 μm). **B** Mean fluorescence intensities of 4T1 cells by flow cytometry. **C** CLSM images of intracellular reactive oxygen species (ROS) (scale bar = 20 μm). **D** Mean fluorescence intensities. All data are mean ± SD (n = 3), statistical significances were calculated via Student’s t test, *p < 0.05, **p < 0.01

Considering the use of MPSNs in biotherapy, it is necessary to evaluate their biosafety and biocompatibility. The viabilities of 4T1 cells were measured after treatment with MPSNs at different concentrations (from 6.25 to 200 μg/mL) by performing MTT assays. As expected, no significant decrease in cell viability occurs after 24 h or 48 h co-incubation, and all results indicate that the MPSNs are almost non-toxic (Additional file 1: Fig S4). To investigate the biocompatibility and endocytosis of MPSNs in cells, 4T1 cells were co-incubated with MPSNs for various times (1, 4, 8, or 24 h), and the intracellular distributions and fluorescence intensities of MPSNs were interrogated by confocal laser scanning microscopy (CLSM) and flow cytometry, respectively. As illustrated in Fig. 3A, bright red fluorescence was observed in 4T1 cells after 4 h co-incubation. Interestingly, the fluorescence intensity is still strong even after 24 h co-incubation, indicating that the MPSNs were rapidly endocytosed by 4T1 cells and remain in cells for 24 h without obvious efflux, which was confirmed by flow cytometry (Fig. 3B). The efficient uptake and low efflux demonstrate that the MPSNs have good biocompatibility, which ensures the high cellular accumulation of MPSNs and enables the intracellular PDT and PTT effects to be realized under laser irradiation.

Furthermore, intracellular ROS generation was studied in 4T1cells by CLSM. The ROS-sensitive probe 2′,7′-dichlorofluorescin diacetate (DCFH-DA) was used to measure ROS levels, based on the rapid oxidation of the nonfluorescent DCFH molecule into the fluorescent molecular dichlorofluorescein in the presence of ROS. As illustrated in Fig. 3C, a stronger green fluorescence signal in the MPSNs (+) and MPSNs@R837 (+) groups was observed after irradiation (0.6 W/cm^2^), whereas nearly no fluorescence was observed both in the PBS groups and in the non-irradiated groups. The mean fluorescence intensities of the MPSNs (+) and MPSNs@R837 (+) groups are significantly higher than those of the other four reference groups (Fig. 3D). Therefore, both the CLSM and flow cytometry experiments confirm the high generation of singlet oxygen in the MPSNs (+) and MPSNs@R837 (+) groups.

In subsequent experiments, the antitumor effect of MPSNs@R837 was further studied in vitro. Bare MPSNs were intrinsically nontoxic to 4T1 cells even at concentrations as high as 250 μg/ mL, which is consistent with the results of previous cytotoxicity experiments. However, irradiated bare MPSNs inhibited 4T1 cells growth in a concentration-dependent manner, due to laser-induced PTT and PDT (Additional file 1: Fig S5A). Next, the effects of free R837 and MPSNs@R837 (with or without laser irradiation) on 4T1 cell growth were evaluated. Additional file 1: Fig S5B shows that MPSNs@R837 (−) and free R837 (with or without irradiation) moderately inhibit 4T1 cell growth, consistent with previous observations that the immune adjuvant R837 can both induce DC maturation and kill cancer cells directly. Distinctly, MPSNs@R837 with irradiation exhibit the highest killing efficiency at a much lower R837 concentration, indicating that the combination of laser-induced PTT and PDT with R837 treatment can significantly improve the antitumor effect. It’s noteworthy that low-power irradiation shows no obvious toxicity to the cells (Additional file 1: Fig S5C). The IC_50_ value of MPSNs@R837 (+) is the lowest among all groups (Additional file 1: Fig S5D), consistent with the above results. Meanwhile, Additional file 1: Fig S6 shows more rapid and extensive cell death for MPSNs (+) or MPSNs@R837 (+) treatment than for other groups, which was consistent with previous results.

To evaluate the effect of PDT without the influence of PTT, an icebox was used to keep the cells at a temperature below 10 °C to eliminate the effect of PTT. Under this condition, the 4T1 cell viability is approximately 65.5%. Afterwards, to evaluate the effect of PTT without the influence of PDT, 4T1 cells were pre-incubated with the ROS inhibitor N-acetylcysteine to quench intracellular ROS, which is generated by laser irradiation. The viability of the 4T1 cells is 59.6% following treatment with PTT alone. As expected, after combined treatment with PTT and PDT, the cell viability decreases sharply, down to as low as 40.3%. In addition, the toxicity of unirradiated MPSNs to 4T1 cells is negligible (Additional file 1: Fig S7).

**Fig. 4:**
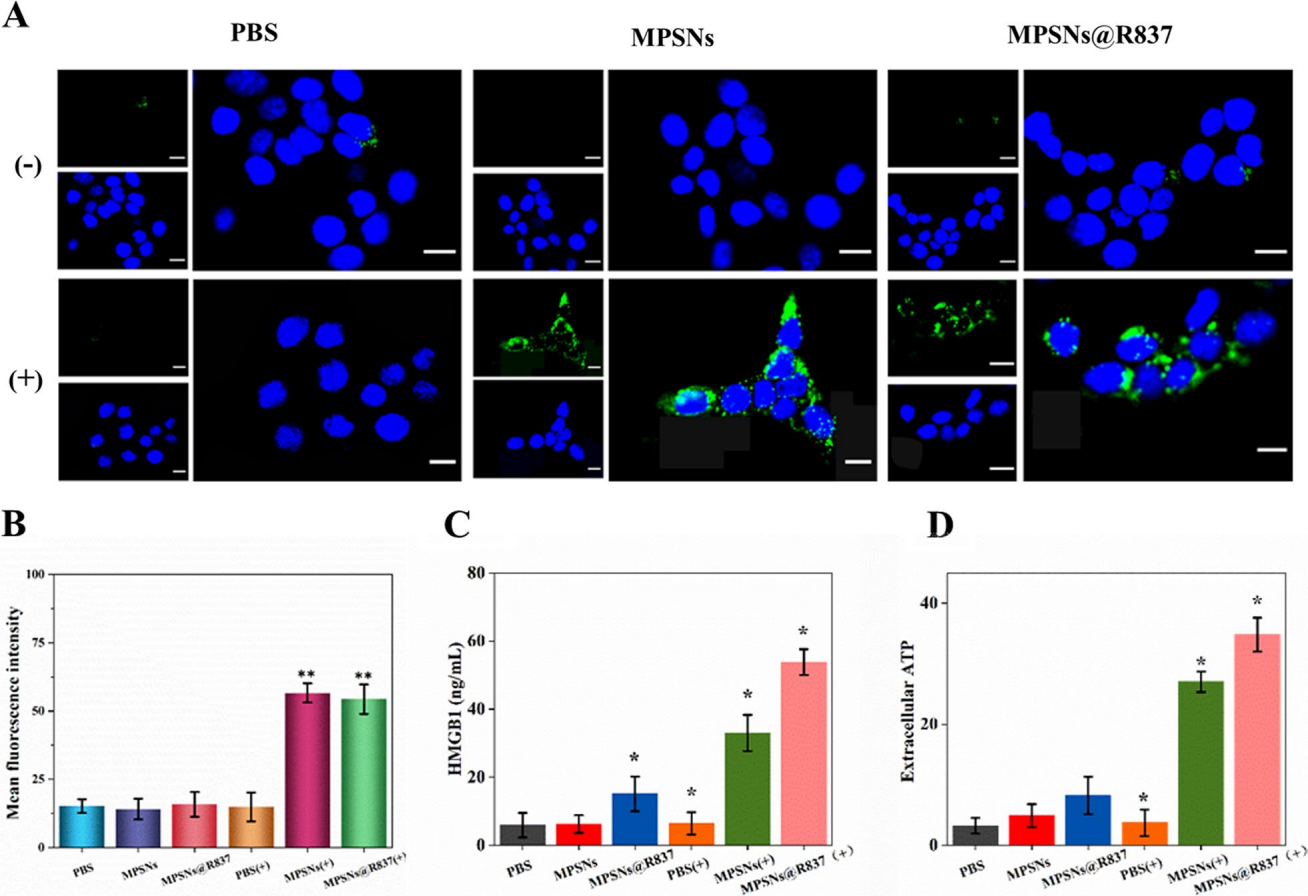
An Immunofluorescence observation of CRT (green fluorescence) exposure on the 4T1 cells surface after incubation with PBS, MPSNs and MPSNs@R837 with or without laser irradiation (808 nm, 0.6 W/cm^2^) (scale bar = 25 μm). **B** Mean fluorescence intensities of 4T1 determined by flow cytometry. **C** HMGB1 and **D** ATP levels of 4T1 cells after 24 h. All data are mean ± SD (n = 3), statistical significances were calculated via Student’s t test, *p < 0.05, **p < 0.01

It is previously reported that phototherapies such as PDT and PTT could induce ICD by inducing high expression of various DAMPs, thereby, causing effective immune responses. Thus, CRT expression, HMGB1 levels, and ATP release were detected in 4T1 cells (Fig. 4). CRT expression on the surface of 4T1 cells after irradiation was tested by both immunofluorescence and flow cytometry. As shown in Fig. 4A, obvious CRT expression was monitored on 4T1 cells treated with MPSNs or MPSNs@R837 after irradiation. In contrast, cell-surface CRT expression is barely detectable in the PBS (+), PBS (−), MPSNs (−) and MPSNs@R837 (−) groups. Flow cytometry yields the similar results (Fig. 4B). MPSNs (+) and MPSNs@R837 (+) induce significantly higher levels of extracellular HMGB1 and ATP, compared to those in all other groups (Fig. 4C, D). The observations of upregulated CRT expression and increased HMGB1 and ATP levels demonstrate that MPSNs@R837 can promote ICD upon irradiation.

**Fig. 5:**
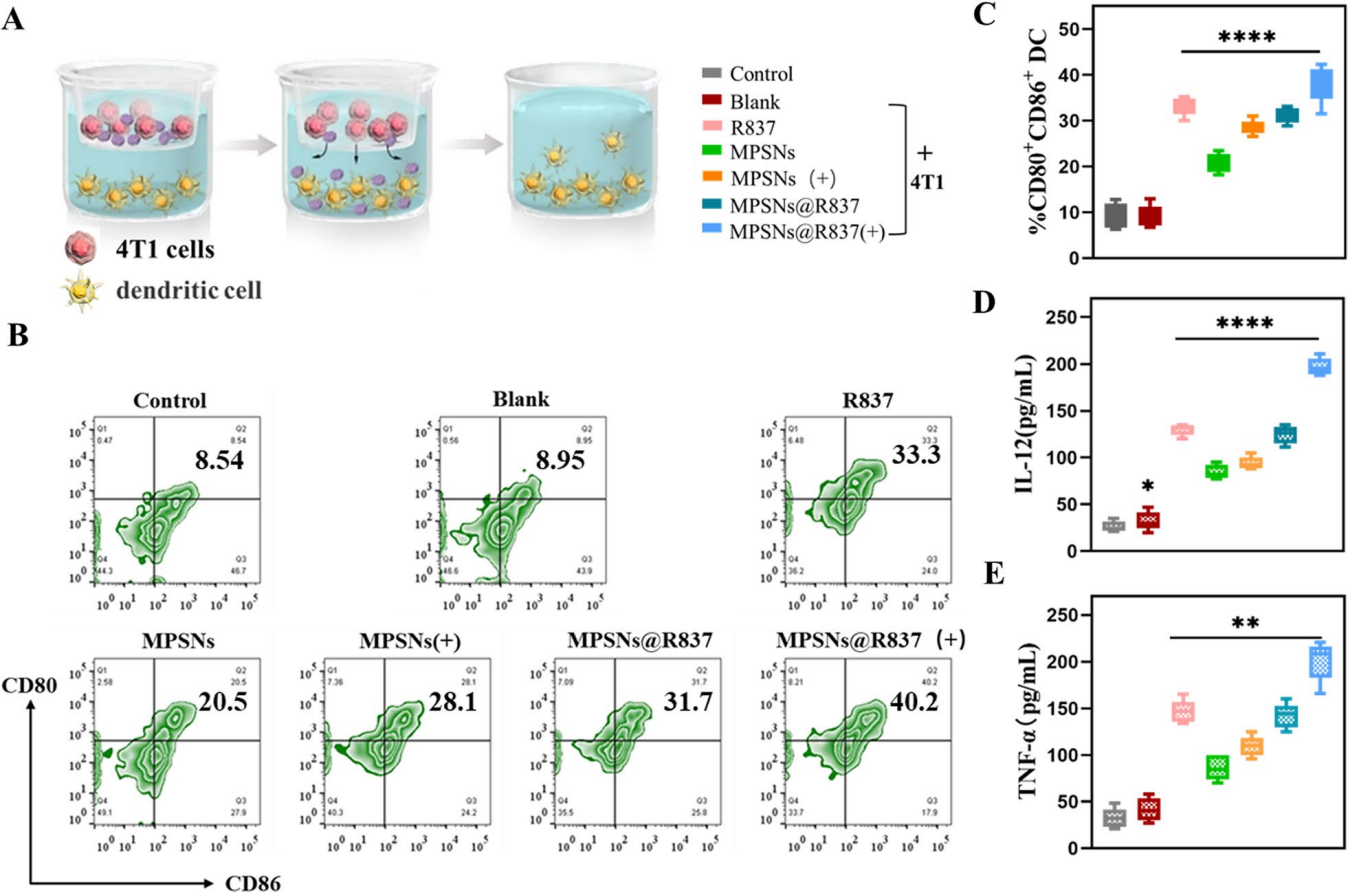
Maturation and secretion analysis of DCs after treated with laser-treated 4T1 cells in the presence of free R837, MPSNs and MPSNs@R837. **A** The design of the transwell system experiment, 4T1 cells were placed in the upper chamber and DCs were incubated in the lower chamber. **B** The expression level of DC maturation (CD11c + CD80 + CD86 +) was determined by flow cytometry after different treatments. **C** Percentage of DC maturation. **D** the secretion of IL-12 and (**E**) TNF-α in DCs suspensions. All data are mean ± SD (n = 3), statistical significances were calculated via Student’s t test, *p < 0.05, ** p < 0.01, *** p < 0.001, **** p < 0.0001

Thereafter, as an immune adjuvant which could stimulate immune responses, the function of the R837 component in MPSNs@R837 was further investigated. The effects of laser-triggered MPSNs@R837 on exciting DC maturation were demonstrated using a transwell system in vitro (Fig. 5). 4T1 cells (after different treatments) and DCs were cultured in the upper and lower chambers, respectively. The extent of DC maturation and secretion of related cytokines were detected by flow cytometry and ELISA, respectively. As expected, the expression of DCs (CD11 + CD80 + CD86 + cells) from 4T1 cells treated with MPSNs@R837(+) is much higher than that in the other groups (Fig. 5B, C), indicating that damaged tumor cells combined with the immune adjuvant R837 could effectively promote DC maturation. In addition, the levels of cytokine secretion (IL-12 and TNF-α) are consistent with DC maturation results, indicating that the laser-irradiated MPSNs@R837 enhanced immune responses (Fig. 5D, E). Collectively, the results presented above show that tumor-associated antigens derived from damaged 4T1 cells (treated with R837-containing nanoparticles as an immune-stimulating adjuvant), could further accelerate DC maturation, potentially triggering a strong immune response.

**Fig. 6:**
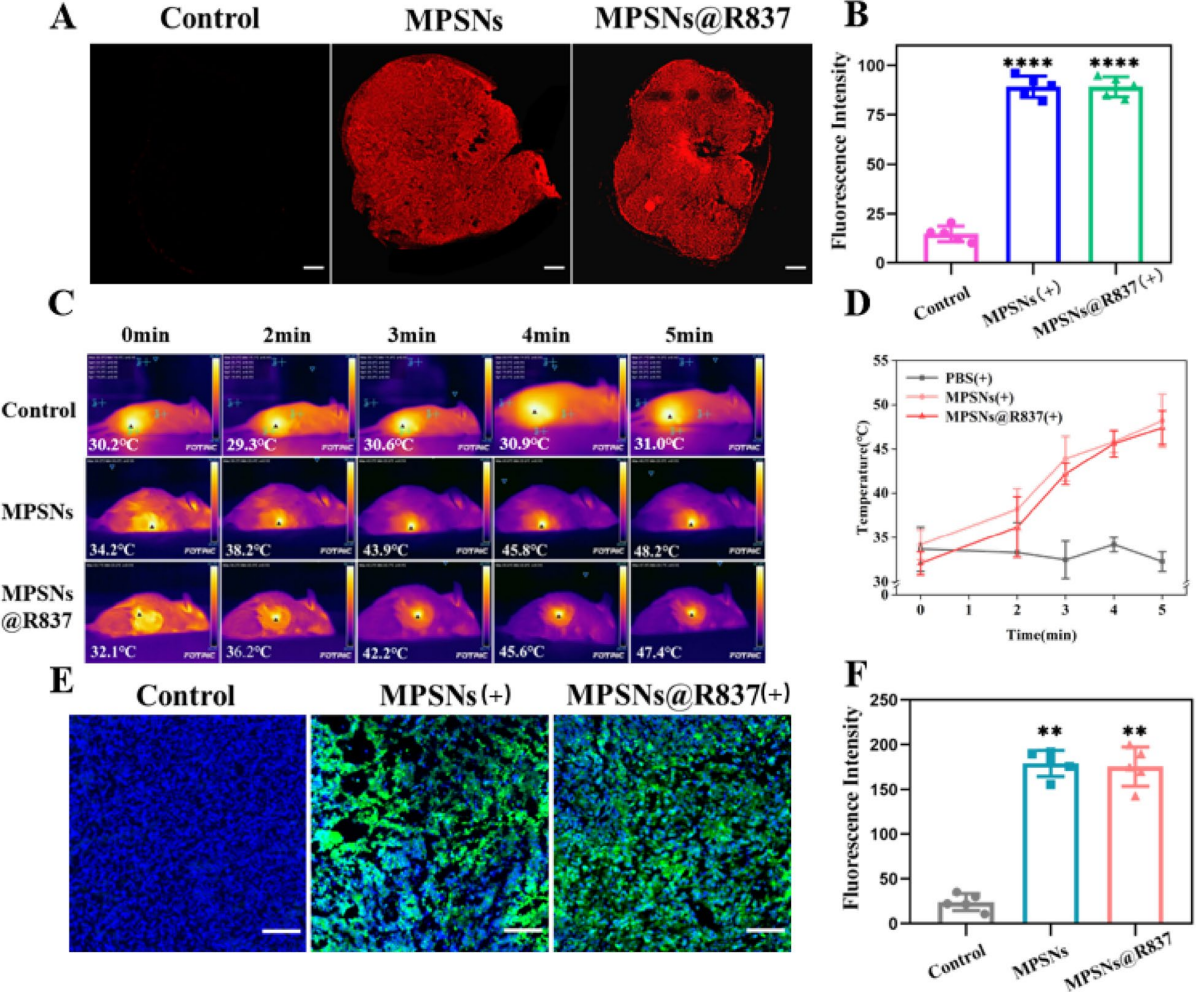
**A** The distribution of MPSNs@R837 (red fluorescence) in tumor sections (scale bar = 200 μm). **B** The fluorescence intensity as described in **A** determined by flow cytometry. **C** Thermographic images and (**D)** tumor temperature changes of 4T1 tumor-bearing mice at different time points under laser irradiation 12 h after injection of saline, MPSNs and MPSNs@R837 (808 nm, 0.6 W/cm^2^). **E** The ROS level in (green fluorescence) in tumor sections (scale bar = 100 μm). **F** The fluorescence intensity as described in **e**. All data are mean ± SD (n = 5), statistical significances were calculated via Student’s t test, ****p < 0.0001

Based on the in vitro experiments, MPSNs@R837 were further studied to evaluate their antitumor effects in vivo. Before conducting therapeutic experiments, the accumulation of MPSNs@R837 in tumor tissues was estimated with a thermal imager. 4T1 tumor-bearing mice were intravenously injected with MPSNs@R837 at a R837 dosage of 3 mg/kg, and other groups were injected with equal doses of MPSNs or PBS, after which they were irradiated (808 nm, 0.6 W/cm^2^, 5 min) at different times post-injection. Photothermal images in Additional file 1: Fig S8A, show that the temperatures around the tumor regions reach the maximum after 12 h in the MPSNs and MPSNs@R837 groups, and the nanoparticles are still retained in the tumor even after 48 h. The temperature changes are presented in detail in Additional file 1: Fig S8B. These phenomena indicate that the MPSNs@R837, possessing a long blood-circulation duration and high stability, are efficiently aggregated in the tumors. To further investigate the accumulation of nanoparticles in the tumors, tumor sections from tumor-bearing mice were examined after injection of MPSNs, MPSNs@R837 and PBS. As shown in Fig. 6A, tumor sections from the MPSNs and MPSNs@R837 treated groups displayed much stronger red fluorescence compared to those in the control group, and flow cytometry yields the similar results (Fig. 6B). These findings provide further evidence indicating that the nanoparticles could efficiently accumulate in tumor tissues. Furthermore, the biodistributions of MPSNs@R837 in tumor tissues and major organs were analyzed using an in vivo fluorescence imaging system. The greatest accumulation of MPSNs@R837 in the tumors was appeared at 12 h and 24 h after intravenous injection (Fig S8C), which is consistent with the thermal imaging results. To further explore the photothermal effects of MPSNs in the tumor site, 4T1 tumor-bearing mice treated with MPSNs, MPSNs@R837 and PBS were exposed to laser irradiation for different times. An infrared thermal camera was used to record the resulting tumor-site temperatures. With the MPSNs and MPSNs@R837 groups, it is identified that the temperatures of tumor sites increase significantly after 5 min of laser irradiation, reaching ~ 48.2 ℃ and 47.4 ℃, respectively, whereas the PBS group shows no significant alteration after the same laser irradiation (Fig. 6C, D). ROS levels in the tumors were then further assessed, where 4T1 tumor-bearing mice were injected with different nanoparticles and H_2_DCFDA, followed by laser irradiation for 5 min. It is noticed that the tumor sections from the MPSNs and MPSNs@R837 treated groups exhibit brighter green fluorescence than those from the control group (Fig. 6E, F). These experimental results disclose that MPSNs@R837 can provoke PTT and PDT effects in tumor sites, showing a great potential for antitumor therapy in vivo.

**Fig. 7:**
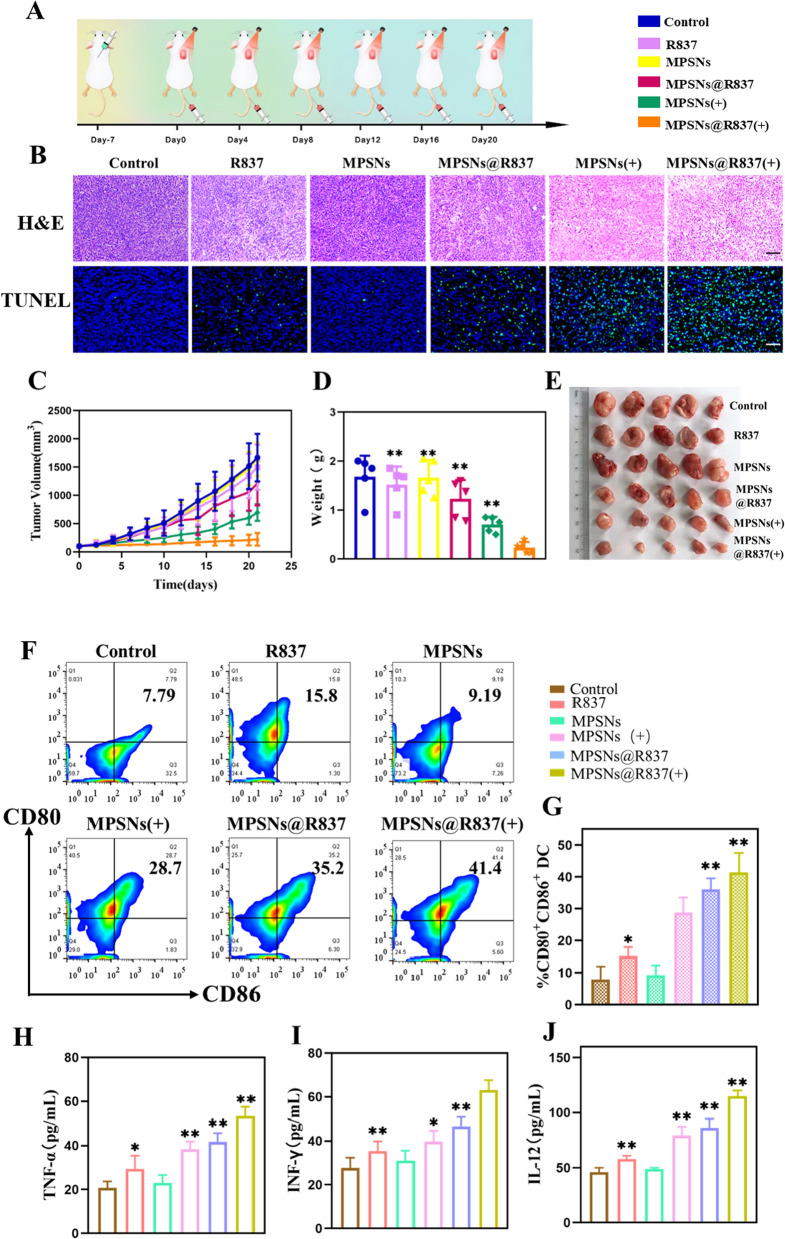
MPSNs@R837-mediated PDT and PTT in vivo: **A** Schematic of treatment schedule in 4T1 orthotropic mammary tumor model. Mice were randomly divided into 6 groups: (1) saline-only, (2) free R837, (3) MPSNs, (4) MPSNs@R837, (5) MPSNs (+), (6) MPSNs@R837 (+). **B** In vivo apoptosis and/or necrosis of the tumor induced by different treatment as shown by H&E staining (scale bar = 100 μm) and TUNEL assay (scale bar = 50 μm). **C** Tumor volume **D** Tumor weight. **E** Tumor picture. **F** and (**G)** DC maturation in the tumor-draining lymph nodes induced by different treatment on mice. **H–J** cytokine levels of TNF-α, INF-γ and IL-12 in sera from mice. Data are mean ± SD (n = 5), statistical significances were calculated via Student’s t test, *p < 0.05, **p < 0.01

Encouraged by the in vivo results, the therapeutic efficacy of MPSNs@R837 was further evaluated in 4T1 tumor-bearing mice (Fig. 7A–E). H&E and TUNEL staining images show that the tumor cells in the MPSNs@R837 group have the highest apoptosis rate (Fig. 7B). As illustrated in Fig. 7C–E, MPSNs@R837 (+) treatment has the greatest inhibitory effect on tumor growth among all groups performed. In addition, free R837, MPSNs (+) and MPSNs@R837 moderately suppress tumor growth, and MPSNs alone elicit no significant inhibitory effect. Afterward, to verify whether MPSNs@R837 (+) can promote DC maturation and immune cytokines secretion, the DC maturation level in draining lymph nodes and serum inflammatory cytokines levels were tested by flow cytometry and ELISA. As expected, MPSNs@R837 (+) facilitate much higher DC maturation (41.4%) compared to that in the other groups (Fig. 7F, G). Taken together, these findings show that in the presence of the immune adjuvant R837, tumor-associated antigens from tumors destroyed by laser can effectively promote DC maturation. Serum inflammatory cytokines (TNF-α, IFN-γ, and IL-12) from 4T1-tumor-bearing mice generally increase after different treatments. Particularly, the cytokine secretions induced by MPSNs@R837 (+) are the highest among all groups (Fig. 7H–J), exhibiting that MPSNs@R837 (+) are helpful to trigger the immune responses. These results verify that laser-irradiated MPSNs@R837 could effectively inhibit tumor growth and elicit immune responses. Importantly, it is essential to assess the biosafety of MPSNs@R837-mediated therapy, therefore, mouse body weights, serum biochemistry and organ histology (liver, spleen, kidneys, heart and lungs) were analyzed (Additional file 1: Figs. S9, S10). All results reveal no significant changes in those parameters, demonstrating the excellent biosafety profile of MPSNs@R837-based treatments.

**Fig. 8:**
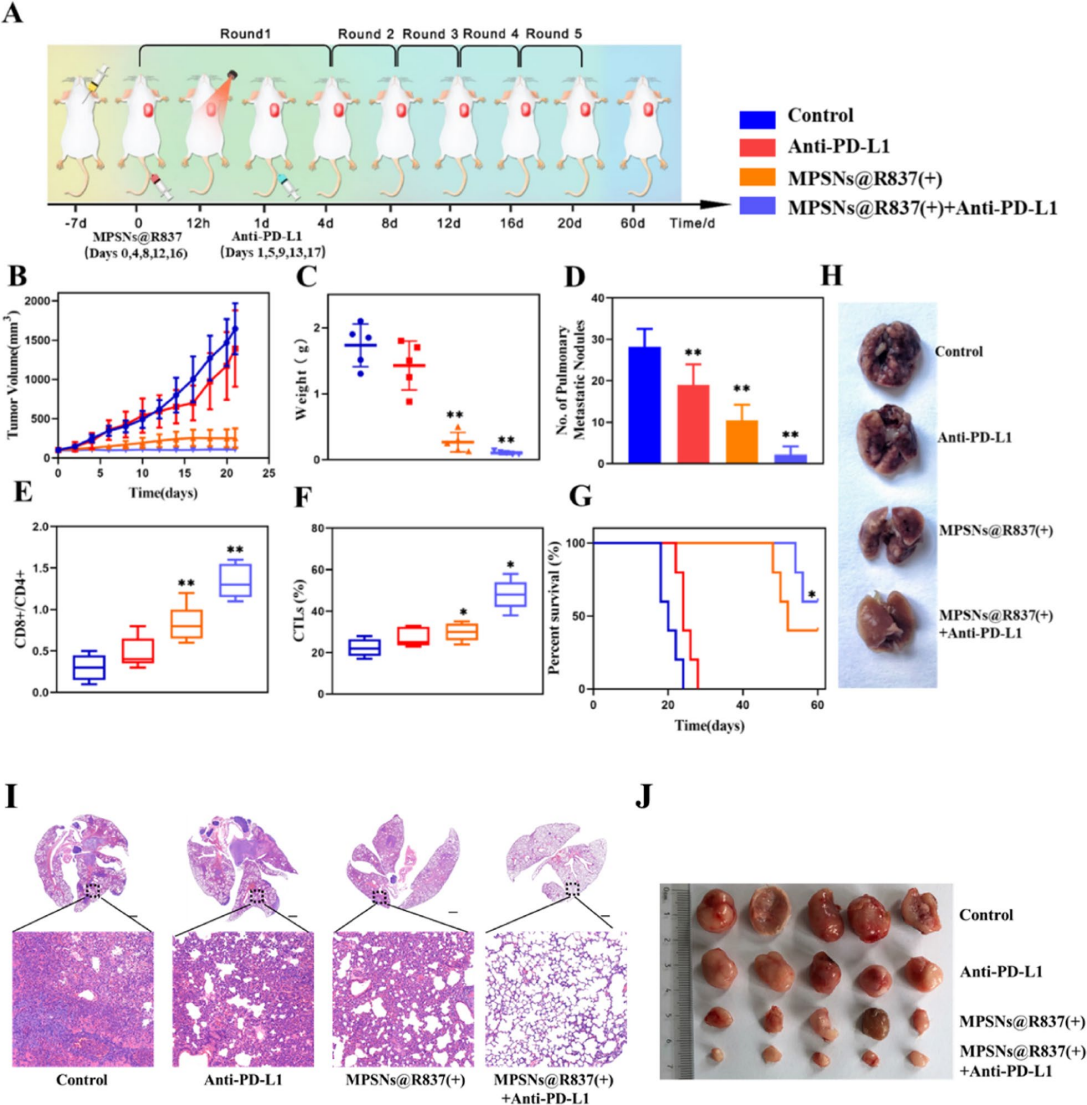
MPSNs@R837-mediated photo-immunotherapy in vivo: **A** Schematic of treatment schedule in 4T1 orthotopic mammary tumor model. Mice were randomly divided into 4 groups: (1) saline, (2) Anti-PD-L1, (3) MPSNs@R837 (+), (4) MPSNs@R837 (+) plus Anti-PD-L1. **B** Tumor volume (**C)** Tumor weight. **D** Number of pulmonary metastatic nodules. **E** Ratio of CD8 + /CD4 + T cells and (**F)** CTL infiltration in primary. **G** Survival time. **H** Lung tissues with metastatic nodules from 4T1 tumor-bearing mice in each group over 21 days. **I** Representative metastatic lung images of each group in Fig. 8. **H**. **J** Tumor images. Data are mean ± SD (n = 5), statistical significances were calculated via Student’s t test, *p < 0.05, **p < 0.01

To further study the therapeutic effects and tumor-specific immune responses of MPSNs@R837, we combined this treatment with a PD-L1 immune checkpoint inhibitor, and evaluated the systemic antitumor ability and anti-metastatic effect (Fig. 8). Notably, MPSNs@R837 (+) plus anti-PD-L1 treatment show a stronger antitumor effect than the other treatments, whereas MPSNs@R837 (+) or anti-PD-L1 treatment alone will not inhibit tumor growth significantly (Fig. 8B, C, J; Additional file 1: Fig S11A). Figure 8D, H show a direct effect against lung metastasis. Surprisingly, the number of lung nodules from mice in the combined treatment group (MPSNs@R837 (+) plus anti-PD-L1) significantly decreases in comparison with those from the monotherapy groups (MPSNs@R837 (+) or anti-PD-L1). Similar results were obtained when performing H&E staining assays (Fig. 8I), demonstrating that the combined treatment strategy possesses a strong ability to inhibit pulmonary metastasis. It is noticed that the CD8 + CD4 + T cells rations, cytotoxic T lymphocyte (CTL) infiltration, and the levels of proinflammatory cytokines (TNF-α, IFN-γ, and IL-12) increase in the MPSNs@R837 (+) plus anti-PD-L1 group (Fig. 8E, F; Additional file 1: Fig S11B–D). In addition, the survival time of mice exposed to combination treatment are significantly prolonged, and half of them survived for 60 days (Fig. 8G). These results certify that photo-immunotherapy with MPSNs@R837 (+) and anti-PD-L1 exerts a greater systemic therapeutic effect in suppressing the growth of primary tumors and pulmonary metastasis. It is noteworthy that MPSNs@R837-mediated photo-immunotherapy will not significantly affect body weights and serum biochemical parameters (Additional file 1: Fig S12). In addition, MPSNs@R837-mediated photo-immunotherapy shows no evident damage to the major organs (Additional file 1: Fig S13), indicating the histocompatibility of anti-PD-L1 plus MPSNs@R837-mediated therapy.

**Fig. 9:**
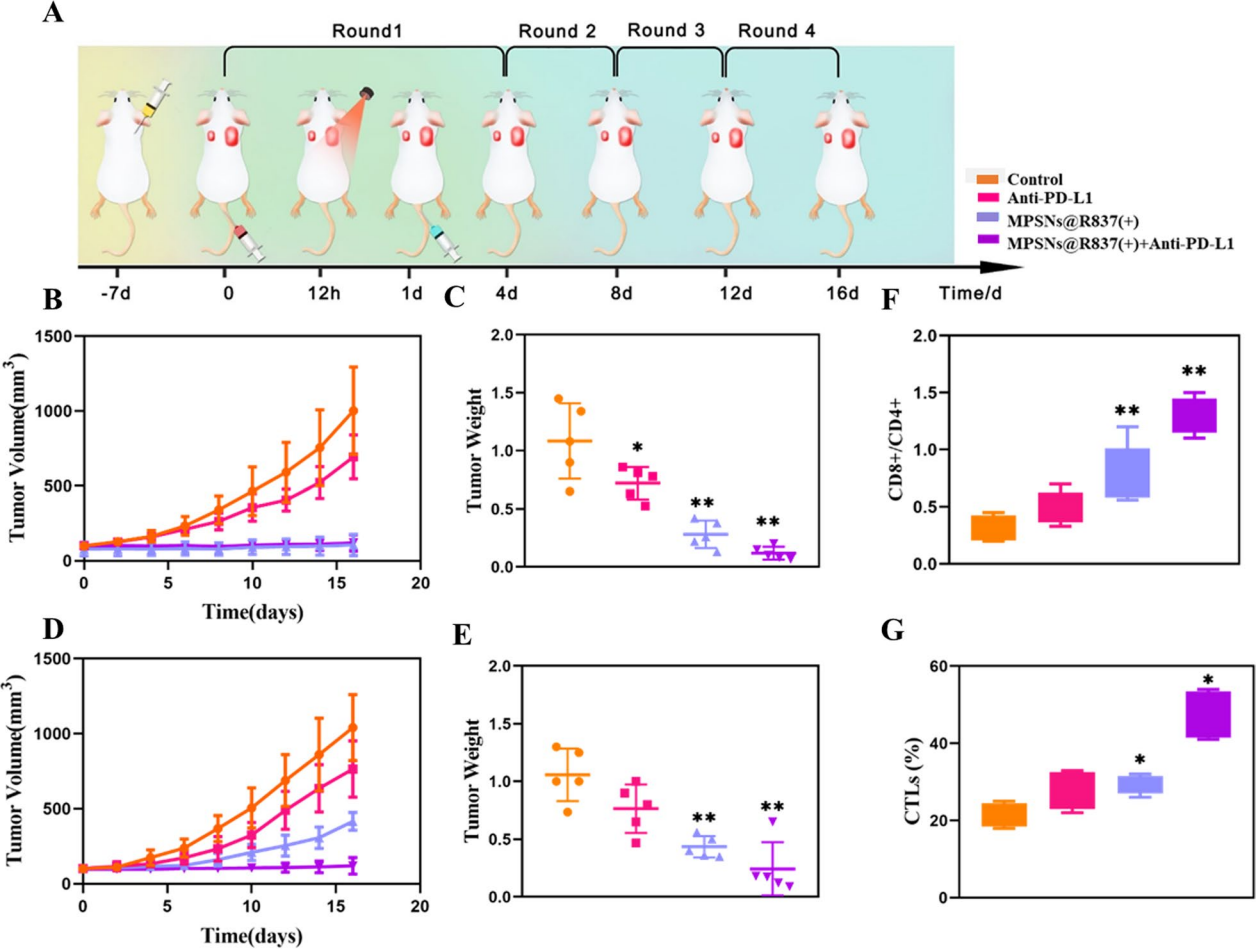
The abscopal effect of MPSNs@R837-mediated photo-immunotherapy: **A** Schematic of treatment schedule a 4T1 bilateral tumor model. Mice were randomly divided into 4 groups: (1) saline, (2) Anti-PD-L1, (3) MPSNs@R837 (+), (4) MPSNs@R837 (+) plus Anti-PD-L1. **B** Volume and (**C)** Weight of primary tumors. **D** Volume and **E** Weight of distant tumors. **F** Ratio of CD8 + /CD4 + T cells and (**G)** CTL infiltration in distant tumor. Data are mean ± SD (n = 5), statistical significances were calculated via Student’s t test, *p < 0.05, **p < 0.01

To explore the systemic immune responses elicited by MPSNs@R837-mediated photo-immunotherapy, we employed a bilateral tumor model by inoculating 4T1 tumors on the flanks of mice to study its therapeutic efficacy. The right tumor (with laser irradiation) was denoted as the primary tumor, and the left tumor (without laser irradiation) was designated as a distant abscopal tumor (Fig. 9A). Similar to the results achieved using a pulmonary metastatic model, the primary tumors of all mice treated with MPSNs@R837-mediated photo-immunotherapy or phototherapy dramatically decrease in volume and weight at the end of the treatment period compared with those in the anti-PD-L1 group. The growth of distant tumors is remarkably inhibited after MPSNs@R837-mediated photo-immunotherapy, but the mice treated with MPSNs@R837-mediated phototherapy still exhibit a rapid growth rate with distant tumors (Fig. 9B–E; Additional file 1: Fig S14A–C), demonstrating that the abscopal effect of therapy without anti-PD-L1 is limited. Subsequently, the mechanism of MPSNs@R837-mediated photo-immunotherapy was carried out by detecting the infiltrating T cells levels in distant tumors and proinflammatory cytokines levels in the serum. The increased CD8 + CD4 + T cells ratio in the photo-immunotherapy group indicate that the tumors are infiltrated of by CTLs (Fig. 9F, G). These results confirmed that MPSNs@R837 (+) plus anti-PD-L1 treatment can promote the tumor infiltration of CD8 + T cells. Interestingly, immunofluorescence imaging of spleens also reveals a distinct enhancement of CD8 + T cells and IFN-γ secretion after MPSNs@R837 (+) plus anti-PD-L1 treatment (Additional file 1: Fig S15). Simultaneously, the serum levels of proinflammatory cytokines (TNF-α, IFN-γ, and IL-12) improve greatly (Additional file 1: Fig S16A–C). There are ignorable pathological changes in terms of body weights, serum biochemical parameters and histopathological staining (liver, spleen, kidneys, heart, and lungs) in the MPSNs@R837-mediated photo-immunotherapy group (Additional file 1: Figs S17, S18).

## Conclusion

In summary, we prepared a novel MPSNs with three functions, PDT, PTT and drug loading, compared with other known nanosystems with relatively limited functions. Usually, integrating the synergistic effect between PDT and PTT requires two photosensitizers with different absorption wavelengths, while MPSNs can efficiently achieve both effects at the same time under only one light source. More notably, MPSNs are not only excellent photosensitizers, but also efficient drug carriers because of the rich mesoporous structures in the silica shell. After loading the immune adjuvant R837, the multifunctional nanoplatform broke through the inhibition of the tumor immunosuppressive microenvironment, effectively promoting DC maturation. The R837-loaded MPSNs efficiently destroyed primary tumors, leading to tumor cell death and promoting the release of tumor-associated antigens, which triggered an immune response by integrating with mature DCs. Furthermore, in combination with PD-L1 checkpoint blockade, photo-immunotherapy could augment PDT and PTT-induced ICD and elicit systemic antitumor immunity, causing regression of primary and metastatic tumors with minimal adverse effects. MPSNs@R837 displayed great potential for combination therapy with PDT, PTT, and immunotherapy, offering a promising and safe photo-immunotherapy strategy for metastatic cancer.

## Supplementary Information

**Additional file 1.** Experimental Section. **Fig S1**. Characterization of MPSNs. **Fig S2**. Fluorescence stability of MPSNs. **Fig S3**. The generation of singlet oxygen of MPSNs determined by the increased SOSG fluorescence. **Fig S4**. Cytotoxicity of MPSNs. **Fig S5**. 4T1 cell viability. **Fig S6**. CLSM images of 4T1 cells stained with Calcein-AM and PI. **Fig S7**. 4T1 cell viabilities after different treatments. **Fig S8**. A Photothermal imaging after intravenous injection of saline, MPSNs and MPSNs@R837 in tumor-bearing mice. B Photothermal heating curves. C The ex vivo fluorescence images of tumors and major organs. **Fig S9**. Representative H&E staining images of heart, liver, spleen, lung, and kidney. **Fig S10**. Change of body weights of mice after different treatments; Serum biochemistry indicators. **Fig S11**. A Tumor volume. B-D cytokine levels of TNF-α, INF-γ and IL-12 in sera from mice. **Fig S12.** Change of body weights of mice after different treatments; Serum biochemistry indicators. **Fig S13**. Representative H&E staining images of heart, liver, spleen, and kidney. **Fig S14**. Tumor volume of A primary and (B) distant tumors of each group. C the images of tumors. **Fig S15**. Representative immunofluorescence staining for CD8a (green) and IFN-γ (red) of spleen sections. **Fig S16**. A-C cytokine levels of TNF-α, INF-γ and IL-12 in sera from mice. **Fig S17**. Representative H&E staining images. **Fig S18**. Change of body weights of mice after different treatments; Serum biochemistry indicators.

## Data Availability

Not applicable.
