# Correction to: Light-triggered multifunctional nanoplatform for efficient cancer photo-immunotherapy

**DOI:** 10.1186/s12951-025-03584-8

**Published:** 2025-07-16

**Authors:** Juan Yue, Qian Mei, Panyong Wang, Peng Miao, Wen-Fei Dong, Li Li

**Affiliations:** 1 School of Biomedical Engineering (Suzhou), Division of Life Sciences and Medicine, University of Science and Technology of China, Hefei, 230026 China; 2 CAS Key Laboratory of Biomedical Diagnostics, Suzhou Institute of Biomedical Engineering and Technology, Chinese Academy of Science (CAS), Suzhou, 215163 China

**Correction to: Journal of Nanobiotechnology (2022) 20:181**

10.1186/s12951-022-01388-8

In this article Fig. 6 appeared incorrectly: the five images in the first row of Fig. 6C used the wrong mouse images, and the temperature values displayed on the images were also incorrect, this error does not affect the scientific conclusions of the paper, so no changes to the main text or conclusion are necessary. For completeness and transparency, the old incorrect and incorrect versions are displayed below.

Incorrect Fig. 6.

**Fig. 6:**
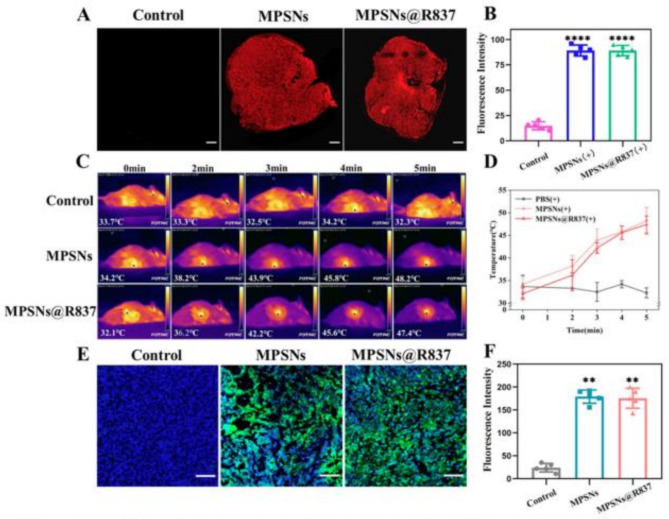
**A** The distribution of MPSNs@R837 (red fluorescence) in tumor sections (scale bar = 200 μm). **B** The fluorescence intensity as described in **A** determined by flow cytometry. **C** Thermographic images and **D** tumor temperature changes of 4T1 tumor-bearing mice at different time points under laser irradiation 12 h after injection of saline, MPSNs and MPSNs@R837 (808 nm, 0.6 W/cm^2^). **E** The ROS level in (green fluorescence) in tumor sections (scale bar = 100 μm). **F** The fluorescence intensity as described in **E**. All data are mean ± SD (*n* = 5), statistical significances were calculated via Student’s t test, ****p < 0.0001

Correct Fig. 6.

**Fig. 6:**
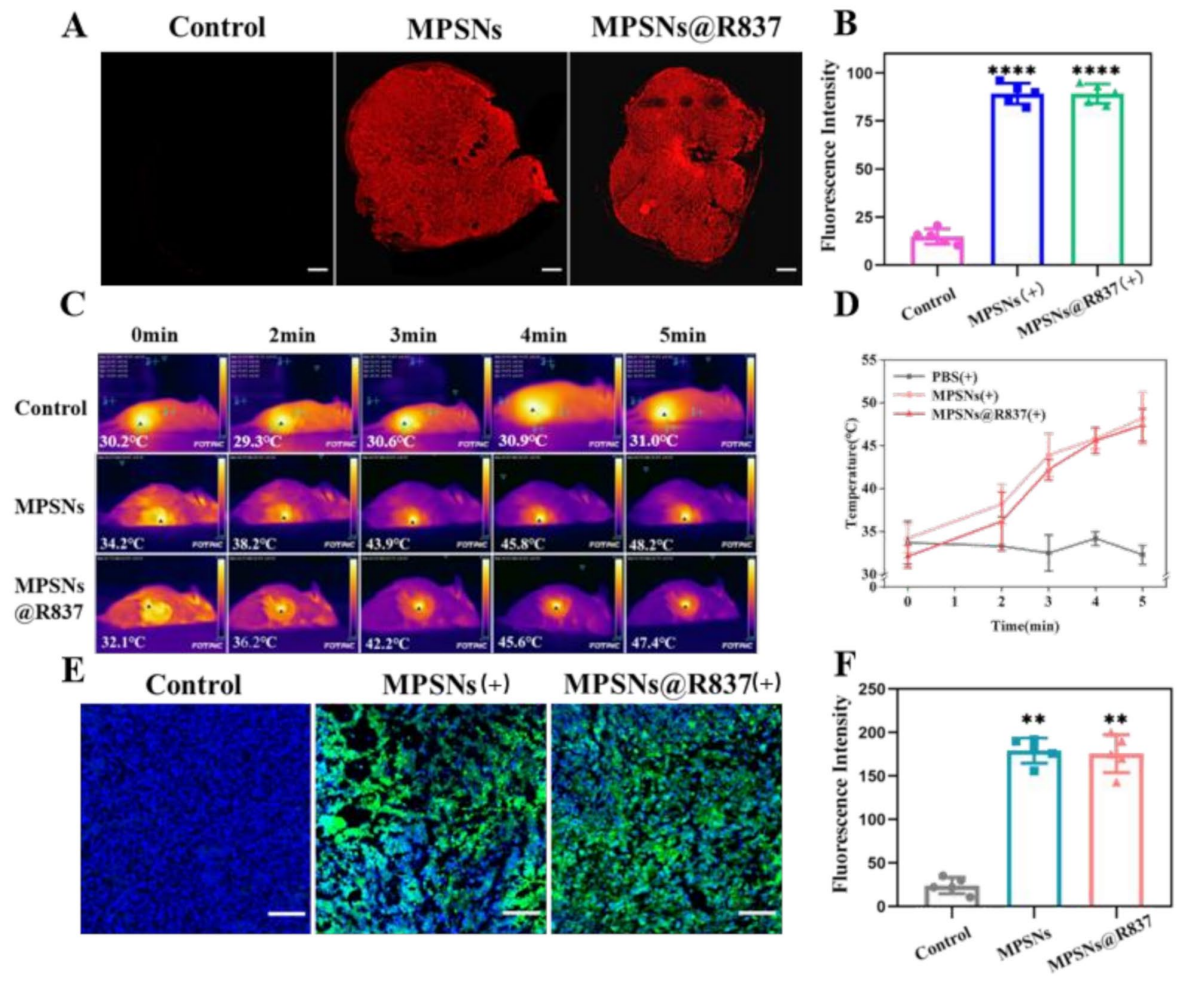
**A** The distribution of MPSNs@R837 (red fluorescence) in tumor sections (scale bar = 200 μm). **B** The fluorescence intensity as described in **A** determined by flow cytometry. **C** Thermographic images and **D** tumor temperature changes of 4T1 tumor-bearing mice at different time points under laser irradiation 12 h after injection of saline, MPSNs and MPSNs@R837 (808 nm, 0.6 W/cm^2^). **E** The ROS level in (green fluorescence) in tumor sections (scale bar = 100 μm). **F** The fluorescence intensity as described in **E**. All data are mean ± SD (*n* = 5), statistical significances were calculated via Student’s t test, ****p < 0.0001

The original article has been corrected.

