# Supplementary material for: Light-triggered multifunctional nanoplatform for efficient cancer photo-immunotherapy
Source: J Nanobiotechnology. 2022 Apr 7;20:181. doi: 10.1186/s12951-022-01388-8 (PMC8991811; doi:10.1186/s12951-022-01388-8)
Supplement: Supplementary file 1 — Additional file 1. Experimental Section. Fig S1. Characterization of MPSNs. Fig S2. Fluorescence stability of MPSNs. Fig S3. The generation of singlet oxygen of MPSNs determined by the increased SOSG fluorescence. Fig S4. Cytotoxicity of MPSNs. Fig S5. 4T1 cell viability. Fig S6. CLSM images of 4T1 cells stained with Calcein-AM and PI. Fig S7. 4T1 cell viabilities after different treatments. Fig S8. A Photothermal imaging after intravenous injection of saline, MPSNs and MPSNs@R837 in tumor-bearing mice. B Photothermal heating curves. C The ex vivo fluorescence images of tumors and major organs. Fig S9. Representative H&E staining images of heart, liver, spleen, lung, and kidney. Fig S10. Change of body weights of mice after different treatments; Serum biochemistry indicators. Fig S11. A Tumor volume. B-D cytokine levels of TNF-α, INF-γ and IL-12 in sera from mice. Fig S12. Change of body weights of mice after different treatments; Serum biochemistry indicators. Fig S13. Representative H&E staining images of heart, liver, spleen, and kidney. Fig S14. Tumor volume of A primary and (B) distant tumors of each group. C the images of tumors. Fig S15. Representative immunofluorescence staining for CD8a (green) and IFN-γ (red) of spleen sections. Fig S16. A-C cytokine levels of TNF-α, INF-γ and IL-12 in sera from mice. Fig S17. Representative H&E staining images. Fig S18. Change of body weights of mice after different treatments; Serum biochemistry indicators. [file 12951_2022_1388_MOESM1_ESM.docx]

**Light-Triggered Multifunctional Nanoplatform for Efficient Cancer** **Photo-Immunotherapy**

Juan Yue^1,2^, Qian Mei^1,2^*, Panyong Wang^1,2^, Peng Miao^1,2^, Wen-Fei Dong^1,2^* and Li Li^1,2^*

*Correspondence: [qmei@sibet.ac.cn](mailto:qmei@sibet.ac.cn), [wenfeidong@sibet.ac.cn](mailto:wenfeidong@sibet.ac.cn), [lil@sibet.ac.cn](mailto:lil@sibet.ac.cn).

^1^School of Biomedical Engineering (Suzhou), Division of Life Sciences and Medicine, University of Science and Technology of China, Hefei 230026, China

^2^CAS Key Laboratory of Biomedical Diagnostics, Suzhou Institute of Biomedical Engineering and Technology, Chinese Academy of Science (CAS), Suzhou 215163, China.

**Experimental Section**

**1. Materials**

Zinc Meso-Tetraphenyl Porphine (ZnP) was purchased from Frontier Scientific, tetraethyl orthosilicate (TEOS), 3-aminopropyltriethoxysilane (APTES), cetyltrimethylammonium bromide (CTAB), imiquimod (R837), ammonium hydroxide and FA-PEG-COOH were purchased from Sigma-Aldrich Co. (St Louis, MO, USA). (RPMI) 1640 medium, fetal bovine serum (FBS), penicillin-streptomycin and 0.25% trypsin-EDTA were purchased from Gibco Co., Ltd. (Carlsbad, CA, USA). Singlet Oxygen Sensor Green reagent (SOSG), Reactive Oxygen Species Assay Kit, WST-1 Cell Proliferation and Cytotoxicity Assay Kit, and DAPI Staining Solution were purchased from Beyotime Biotechnology Co., Ltd. (China). Anti-mouse ELISA kits (IL-12, INF-γ and TNF-α) were bought from MultiSciences (Lianke) Biotechnology Co., Ltd. (China). Anti-PD-L1 was obtained from Bioxcell (α-PD-L1, Clone: 10 F.9G2, Catalog No. BE0101). Fluorochrome-labeled anti-mouse monoclonal antibodies (anti-CD8a APC, anti-CD3 PerCP/cy5.5, Anti-CD4 FITC, anti-CD11c APC, anti-CD80 PE and anti-CD86 FITC) were purchased from BioLegend, Inc (San Diego, CA, USA). All other reagents were used without further purification.

**2. Preparation of MPSNs**

MPSNs was produced via an improved two-step method.[1] Firstly, 5 mg ZnP was dissolved in 1mL of hydrochloric acid aqueous solution (Hcl: 0.5 mM) and stirred at room temperature for 1 h. Then, the mixture was added to 10 ml aqueous solution including CTAB (10 mM) and NaOH (2.5 mM) with continuous stirring for 24 h at room temperature, the pre-MPSNs were collected. Secondly, 50 mg CTAB was dissolved in 10 mL ultrapure water, the pre-MPSNs were added to the CTAB solution, and then ultrasonic treatment was applied for 30 min. The mixture was stirred in a water bath, then 0.05 mL TEOS and 0.01 ml APS were injected into the solution, followed by 0.01 ml NH_4_OH. The reaction was allowed to proceed 40℃ for 1 h, and the MPSNs- NH_2_ was obtained by centrifugation. Finally, the EDC/NHS method was used to covalent conjugation between FA-PEG-COOH and MPSNs-NH_2_ according to previous reports. In brief, MPSNs-NH_2_ suspension was added to the EDC/NHS aqueous solution and sonicated. Thereafter, FA-PEG-COOH was added to the mixed suspension and reacted for 12 h. The reactants were collected by centrifugation at 8,000 rpm and washed with water three times. The final PEG-conjugated product was named as MPSNs.

**3. MPSNs characterization**

The morphology and structure of MPSNs was characterized by transmission electron microscopy (JEOL Ltd, Japan) with a 200 kV accelerating voltage. Energy-dispersive X-ray spectroscopy (EDS) was determined by a JEM-2100F EDX system. The average hydrodynamic size and the zeta potentials of MPSNs were measured via a Zeta sizer NanoZS (Malvern Instruments, USA). The Brunauer-Emmett-Teller (BET) method was employed to test the surface area and pore size distribution of MPSNs. The UV-Vis adsorption spectral values were recorded through a U-3310 spectrophotometer (Hitachi). Fluorescence spectra was analyzed on a Hitachi 135 F-4600 fluorescence spectrophotometer.

**4. Loading and release of R837 in vitro**

For R837 loading, R837 was dissolved in dilute acetic acid to obtain a stock solution (1 mg/mL). 10 mg MPSNs was suspended in 10 ml deionized water to prepare MPSNs solution, then R837 solution was added to MPSNs solution under stirring at room temperature for 48 h followed by centrifugation to obtain MPSNs@R837. Then, the mixtures were washed and dried for further applications. The adsorption amount of R837 was performed by analyzing the absorbance of the supernatant at 320 nm, and the R837 loading content was calculated according to the equation, drug loading content (%) = mass of R837 in MPSNs@R837/ mass of MPSNs@R837.

2 mL MPSNs@R837 solution (1mg/mL) was added to dialysis bags at different pH values (pH 7.4 or 5.0) respectively and stirred at 37°C. The amount of R837 release was detected by UV absorption spectroscopy.

**5. In Vitro Singlet Oxygen Generation Detection**

Singlet Oxygen Sensor Green (SOSG) was used to detect the ^1^O_2_ generation of MPSNs and MPSNs@R837. Briefly, various samples (MPSNs, MPSNs@R837) in water were added to SOSG solution, and then the samples were irradiated by808 nm laser at the power density of 0.6 W/cm^2^ for different time. Finally, the fluorescence change of SOSG (excitation wavelength: 488 nm; emission wavelength 525 nm) were applied to measure the generated ^1^O_2_.

**6. Photothermal Study of MPSNs**

500 µL MPSNs aqueous solution (100 µg/mL) was irradiated with laser (808 nm, 0.6 W/cm^2^) for 600 s, and the temperature of MPSNs was recorded every 15 s, while deionized water was used as a control. In order to better study the photothermal activity, MPSNs solution with different concentrations (25, 50, 75, 100 µg/mL) were irradiated using 808 nm laser for 600 s (0.6 W/cm^2^). Meanwhile, MPSNs aqueous solution (100 µg/mL) were irradiated with 808 nm laser at various power densities (0.2, 0.4, 0.6, 0.8 W/cm^2^) for 600 s. Moreover, the MPSNs solution was irradiated for 600 s followed by a 600 s cooling period. Such heating-cooling process was repeated for 5 cycles.

**7. Photothermal Conversion Efficiency of the MPSNs**

Photothermal conversion efficiencies of MPSNs were measured according to previous report.[2] The detailed calculation was carried out as following equations:

$\eta=\frac{\mathrm{hA}\left( \mathrm{Tmax}-T\mathrm{surr} \right)-Qdis}{I(1-{10}^{-A808})}$ (1)

Where h and A represent the heat transfer coefficient and the surface area of the container. T_max_ represents the temperature of MPSNs after laser irradiation, T_surr_ is the room temperature. *Q*_dis_ is the heat dissipation of system, and it is calculated to be approximately equal to 0 mW. I is the laser power (0.6 W/cm^2^). A808 is the absorbance of MPSNs at 808 nm. The hA is calculated using the following equation (2)

$\tau_{s}=\frac{m_{D} c_{D}}{\mathrm{hA}}$ (2)

Here τ_s_ is the time constant, which could be determined by linear regression of time in cooling period versus lnθ. It was calculated to be τs = 286.08 from Fig 2d. m_D_ and c_D_ are the mass (500 mg) and heat capacity (4.2 J·g^-1^·℃^-1^) of the solvent. The photothermal conversion efficiency of the MPSNs was listed in the table.

|  | Tmax-Tsurr | A_808_ | τs | η |
| --- | --- | --- | --- | --- |
| CMS | 35.5℃ | 2.06 | 286.08 | 43.8% |

**8.** **Cell Culture**

4T1 cells purchased from Cell Bank of Chinese Academy of Sciences (Shanghai, China). Cytoxicity of MPSNs was evaluated using the MTT assay. Briefly, 4T1 cells were seeded in 96-well plates (5×10^3^ cells/ well) and cultured in RPMI-1640 medium supplemented with 10 % (v/v) heat-inactivated fetal bovine serum with 5% CO_2_ at 37 °C. MPSNs at various concentrations were co-incubated with 4T1 cells for 24 h and 48 h, respectively. Then, the cells were washed with PBS and incubated with MTT (500 μg/mL) at 37 °C for another 3 h. Finally, MTT formazan remained was dissolved with DMSO and the absorbance was detected by a microplate reader. The cell viability of the variously treated groups was calculated.

**9.** **Cellular uptake**

4T1 cells were seeded in cell dishes and were cultured with MPSNs (12.5 μg/ml) for 1 h, 4 h, 8 h and 24 h respectively. The cells were then washed with PBS for three times, and the distribution of intracellular nanoparticles was observed by confocal scanning microscopy (CLSM). At the same time, cellular fluorescence of MPSNs was detected by flow cytometry (FACS, Becton-Dickinson Biosciences, Drive Franklin Lakes, USA). All statistical analyses were based on three independent experiments.

**10. In vitro ^1^O2 generation at the cellular level**

4T1 cells were seeded in confocal small dishes and cultured for 24 h, then they were co-incubated with MPSNs or MPSNs@R837 for 12 h, fluorescent dye H_2_DCFDA (10 μM) was further used to treat all cells for another 30 min. Next, the cells were washed three times and irradiated by 808 nm laser (0.6 W/cm^2^) for 3 min. The cellular ROS were observed by CLSM and FACS.

**11. In vitro cytotoxicity assay**

MTT assay was used to evaluate phototherapeutic toxicity. In short, 4T1 cells were seeded in 96-well plates (5 × 10^3^ cells per well) and incubated in RPMI-1640 medium containing 10% FBS for 24 h (37 °C, 5% CO_2_). Then, the medium was removed and the refreshed RPMI-1640 medium containing different concentrations of free R837, MPSNs or MPSNs@R837 was added to each well and incubated for another 24 h. For R837 (+), MPSNs (+), MPSNs@R837 (+) groups, cells were irradiated with laser light (808 nm, 0.6 W/cm^2^) for 5 min and further incubated for 24 h. All groups were performed in sextuplicate. The ratio of each group to the control group was calculated to determine the IC_50_ value.

**12. CRT Exposure**

Flow cytometry was applied to estimate the CRT exposure on the surface of 4T1 cells. In short, 4T1 cells were co-incubated with MPSNs or MPSNs@R837 for 12 h, which then received irradiation (808 nm, 0.6 W/cm^2^) for 5 min and incubated for 4 h. The cells were washed and incubated with CRT antibodies for 45 min. Next, the samples were analyzed by FACS.

In order to better observe the exposure of CRT on the surface of 4T1 cells, confocal microscopy was used for further observation. Shortly, the cells in confocal small dishes were co-incubated with MPSNs for 12 h, then received laser irradiation or not. Afterwards, the treated cells were stained by CRT antibodies for 45 min and observed by CLSM.

**13. DC stimulation transwell experiment in vitro**

Bone-marrow-derived DCs were isolated from 8-week-old balb/c mice. The transwell mode is a cocultivation system. 4T1 cells were incubated with MPSNs or MPSNs@R837 in the upper compartment, while the DCs were seeded in the lower compartment. DCs stained with anti-CD11c, anti-CD80, and anti-CD86 antibodies were analyzed by flow cytometry after various treatment. Meanwhile, the proinflammatory cytokines (IL-12, TNF-α and INF-γ) in DCs medium suspensions were determined by ELISA kits following standard protocols.

**14. Animal experiments**

Female balb/c mice (6-8 weeks, 18-20g) were purchased from Beijing Institution for Drug Control, China. All animal experiments were strictly performed in accordance with the institutional guidelines for animal experimentation and were approved by the Ethics Committee for the Use of Experimental Animals of the Suzhou Institute of Biomedical Engineering and Technology, Chinese Academy of Sciences (Suzhou, Jiangsu, China).

**15. Biodistribution**

MPSNs@R837 (3 mg/kg R837) was intravenously injected into female balb/c mice bearing the 4T1 tumors. The mice were imaged with infrared thermographic camera (Fotric, USA) at the time points of 0, 8th, 12th, 24th and 48th hour postinjevtion after irradiated with red light (808 nm, 0.6 w/cm^2^, 5min), respectively. Twenty-four hours and forty-eight hours after injection, the mice were sacrificed, and the main organs (liver, spleen, kidneys, heart and lungs) and tumors were collected. These organs were then imaged with an IVIS Spectrum (PerkinElmer).

**16. Measurement of ROS *in vivo***

4T1 bearing mice were tail vein injection fluorescent dye H_2_DCFDA (10 mM).[3] Then MPSNs and MPSNs@R837 were intravenously injected into mice and irradiated after 12 h injection (808 nm, 0.6 W/cm^2^, 5 min). Finally, the tumors were collected to make frozen sections and observed by CLSM.

**17. Anti-tumor activity of MPSNs *in vivo***

Female balb/c mice (6-8 weeks) were randomly divided into six groups (n=5), including: (1) control, (2) R837, (3) MPSNs, (4) MPSNs@R837, (5) MPSNs (+), (6) MPSNs@R837 (+). 4T1 cells (5 × 10^5^) were subcutaneously injected into the right shoulder of each mouse. The experiment started when the tumors were allowed to 100 mm^3^. Each group was intravenously injected R837, MPSNs, MPSNs@R837 (R837 dose of 3 mg/kg) or saline with the same amount. For MPSNs (+) and MPSNs@R837 (+) groups, mice were irradiated with laser light (808 nm, 0.6 w/cm^2^, 5 min) after 12 h injection. Tumor volume and body weight were evaluated every 2 days. Tumor volume was calculated as V= L×W^2^/2 (mm^3^), where L is the longest dimension and W is the shortest dimension, respectively. Three days later, tumors were collected and stained with H&E and TUNEL assay. The tumor-draining lymph node cells were isolated to determine the DC maturation by FACS after staining with anti-CD11c APC, anti-CD80 PE and anti-CD86 FITC. In addition, blood samples were collected from mice after different treatments, and the proinflammatory cytokines (IL-12, TNF-α and INF-γ) were detected by ELISA.

**18. Anti-tumor activity and immune response**

4T1 cells (5×10^5^) were orthotopically injected into the mammary fat pads of each mouse. Mice were randomly divided into 4 groups (n=5), including (1) control, (2) Anti-PD-L1, (3) MPSNs@R837 (+), (4) MPSNs@R837 (+) plus Anti-PD-L1. Then mice were i.v. injected saline, Anti-PD-L1, MPSNs@R837 or MPSNs@R837 plus Anti-PD-L1 respectively (equal R837 dose of 3 mg/kg) every 4 days. Anti-PD-L1 at the dose of 75 μg/mouse was administered on days 1, 5, 9, 13 and 17. The tumors of MPSNs@R837 (+) and MPSNs@R837 (+) plus Anti-PD-L1 groups were irradiated after 12 h injection (808 nm, 0.6 W/cm^2^, 5 min). Similarly, tumor volume was monitored during the duration. To better evaluate the immune response *in vivo*, the mice were sacrificed to collect tumor and serum on the 6th day after treatment, the tumors were prepared into a single-cell suspension with a tissue dissociation kit. The harvested lymphocyte cells were stained with anti-CD8a APC, anti-CD3 PerCP/cy5.5, Anti-CD4 FITC antibodies and then analyzed by FACS to detect the CTLs (CD3^+^CD4^-^CD8^+^) and CD4^+^ T cells (CD3^+^CD4^+^CD8^-^), meanwhile the INF-γ, TNF-α and IL-12 were detected by ELISA. The mice were sacrificed after 21 days of treatment. The liver, lung, heart, spleen, and kidney were collected, fixed and stained with H&E. Biochemical parameters, including AST, ALT, BUN and CREA, were tested by Coulter LX2D instrumentation (Beckman, Brea, CA). Lung tissues were imaged and the number of metastatic nodules was recorded.

In a parallel experiment, 4T1 tumor-bearing mice were treated as described above, and overall survival was recorded within 60 days. When the tumor volume exceeded 2,000 mm^3^ or the health of the mouse was significantly impaired, mice were euthanized and recorded as death.

**19. Anti-tumor activity and immune response in bilateral 4T1 orthotopic mammary tumor-bearing mice**

4T1 cells (5 × 10^5^) were subcutaneously injected into the right flank of each mouse to simulate the primary tumors. Six days later, 4T1 cells were subcutaneously injected into the left of each mouse to simulate the distant tumors. Subsequently, these mice were randomly divided into four groups (n=5), including (1) control, (2) anti-PD-L1, (3) MPSNs@R837 (+), (4) MPSNs@R837 (+) plus anti-PD-L1. Mice were intravenously injected with saline, anti-PD-L1, MPSNs@R837 and MPSNs@R837 plus anti-PD-L1 at the same doses as those mentioned above. The laser irritation was performed after 12h post injection. Tumor volume and body weight were measured every 2 days. On the 7th day after treatment, blood was collected from orbital of mouse to measure IFN-γ, IL-12 and TNF-α. At the same time, T cells infiltration of distal tumors was collected from using the aforementioned method and texted by FACS.

After 16 days of treatment, all mice were sacrificed, and main organs including liver, spleen, kidneys, heart, and lungs were collected, fixed, and stained with H&E, while tumors were excised, weighed and photographed. Biochemical parameters, including AST, ALT, BUN, and CREA were analyzed automatically using Coulter LX2D instrumentation (Beckman, Brea, CA).

To better investigate antitumor immune responses *in vivo*, the spleens were dissected and spleen sections were prepared, then incubated with CD8a or IFN-γ antibody. Finally, sections were stained with DAPI and observed by CLSM.

**Statistical analysis**

All data were representative results from at least three independent experiments and means ± SEM. The correlation and comparison analyses were performed using the student’s t-test. *p < 0.05 was considered a statistically significant difference.

**References**

1. Wang Z, Shao D, Chang Z, Lu M, Wang Y, Yue J, Yang D, Li M, Xu Q, Dong WF: **Janus Gold Nanoplatform for Synergetic Chemoradiotherapy and Computed Tomography Imaging of Hepatocellular Carcinoma.** *ACS Nano* 2017, **11:**12732-12741.

2. Zheng X, Wang L, Liu S, Zhang W, Liu F, Xie Z: **Nanoparticles of Chlorin Dimer with Enhanced Absorbance for Photoacoustic Imaging and Phototherapy.** *Advanced Functional Materials* 2018, **28**.

3. Jiang L, Zhou S, Zhang X, Li C, Ji S, Mao H, Jiang X: **Mitochondrion-specific dendritic lipopeptide liposomes for targeted sub-cellular delivery.** *Nat Commun* 2021, **12:**2390.


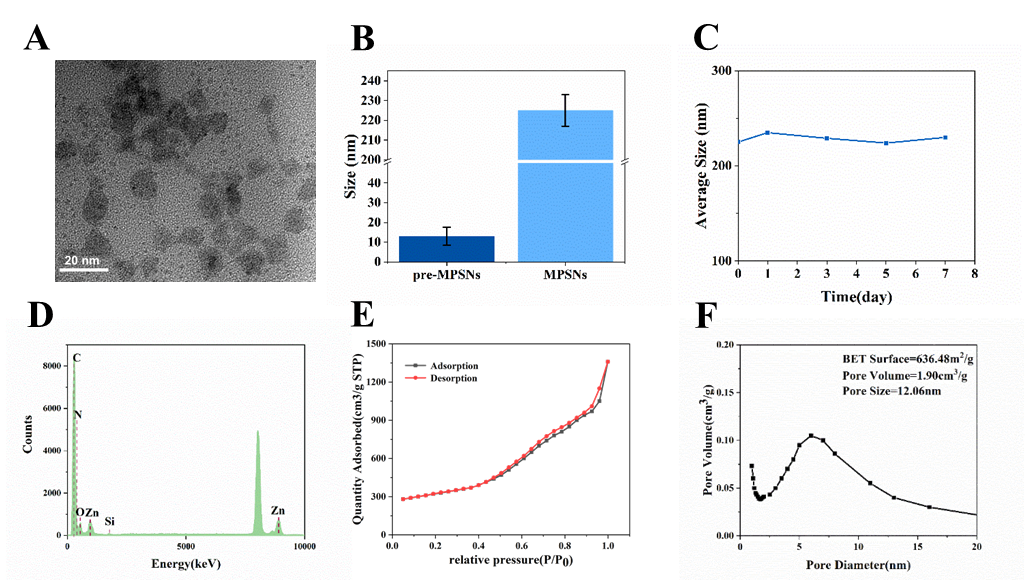


**Figure S1.** Characterization of MPSNs. **A** TEM image of pre-MPSNs. **B** The size distribution of MPSNs and pre-MPSNs in water. **C** Time-dependent colloidal stability of MPSNs. **D** Energy-dispersive X-ray spectroscopy (EDS) spectra of MPSNs. **E** N_2_ sorption isotherms of MPSNs. **F** Pore size distribution of MPSNs.


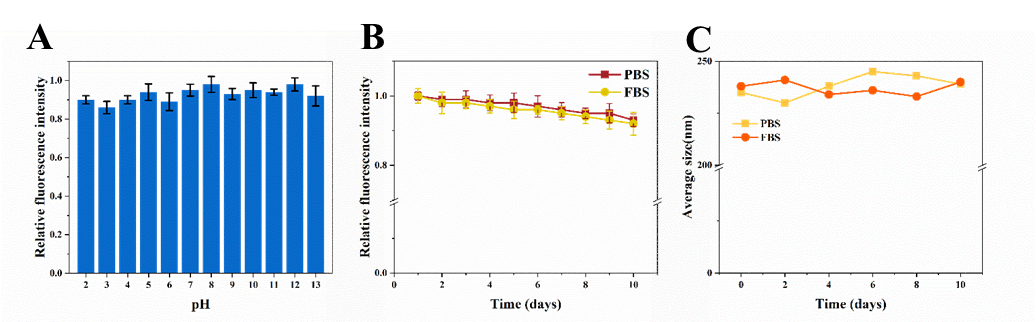


**Figure S2**. **A** Fluorescence stability of MPSNs at different pH values (from 2 to 13). Fluorescence stability (**B)** and colloidal stability (**C)** of MPSNs after 10 days storage in PBS or FBS.


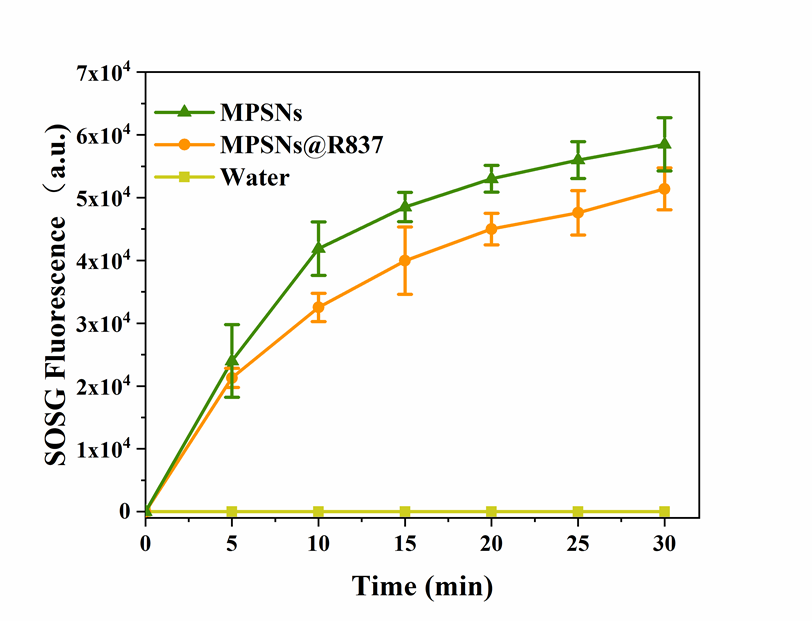


**Figure S3** The generation of singlet oxygen of MPSNs determined by the increased SOSG fluorescence.


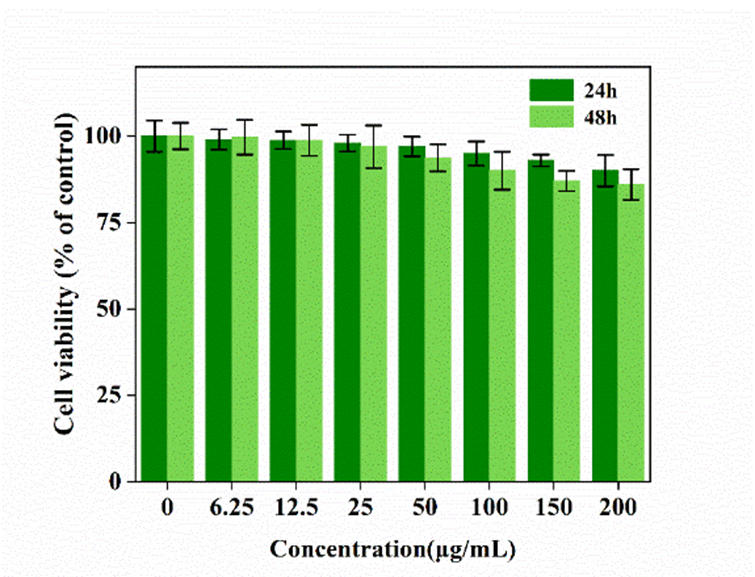


**Figure S4.** Cytotoxicity of MPSNs after 24 h or 48 h incubation.


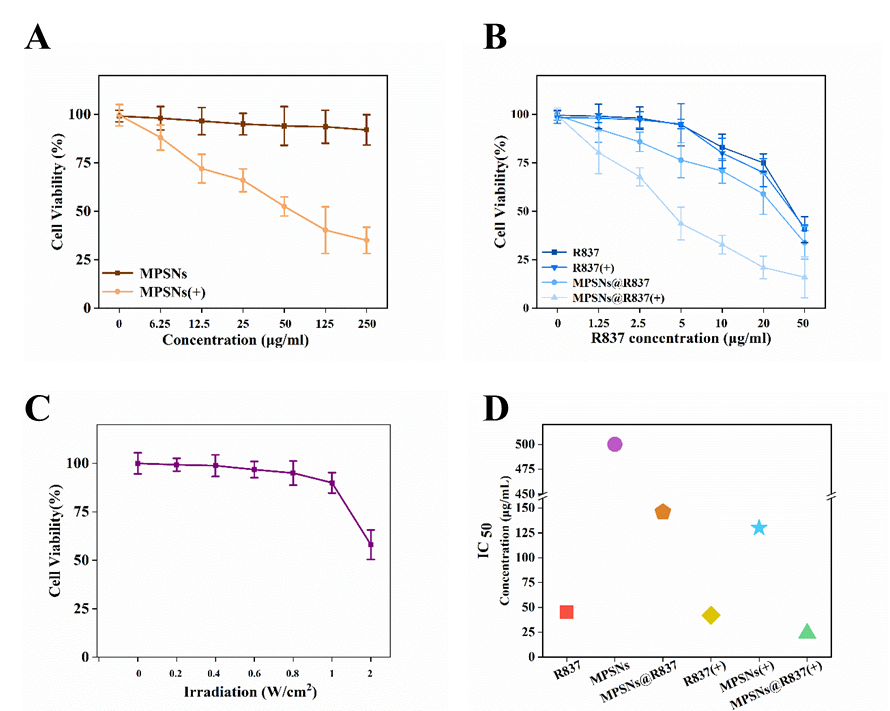


**Figure S5.** 4T1 cell viability following treatment with different doses of (**A**) MPSNs with or without laser irradiation. **B** free R837, MPSNs@R837 with/without laser irradiation. **C** laser radiation at various power densities. **D** IC_50_ of each group after 24 h of exposure.


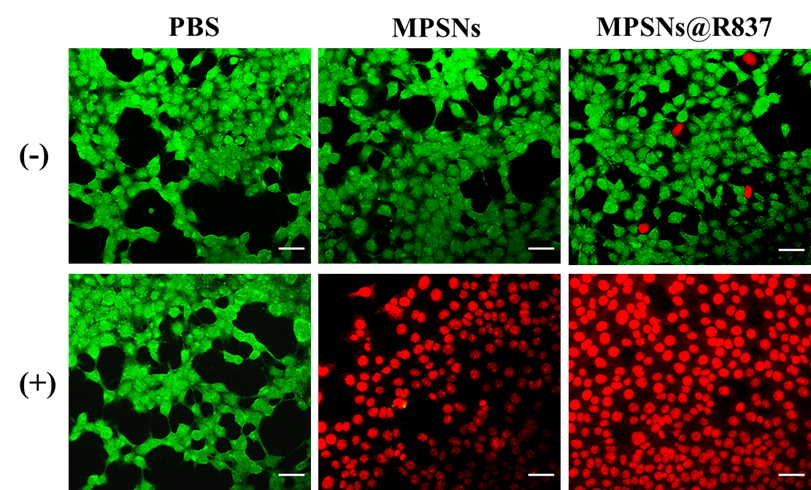


**Figure S6**. CLSM images of 4T1 cells stained with Calcein-AM and PI after various treatments: PBS, MPSNs, MPSNs@R837 (with laser irradiation (+), without laser irradiation (-)) (scale bar = 50 μm).

*
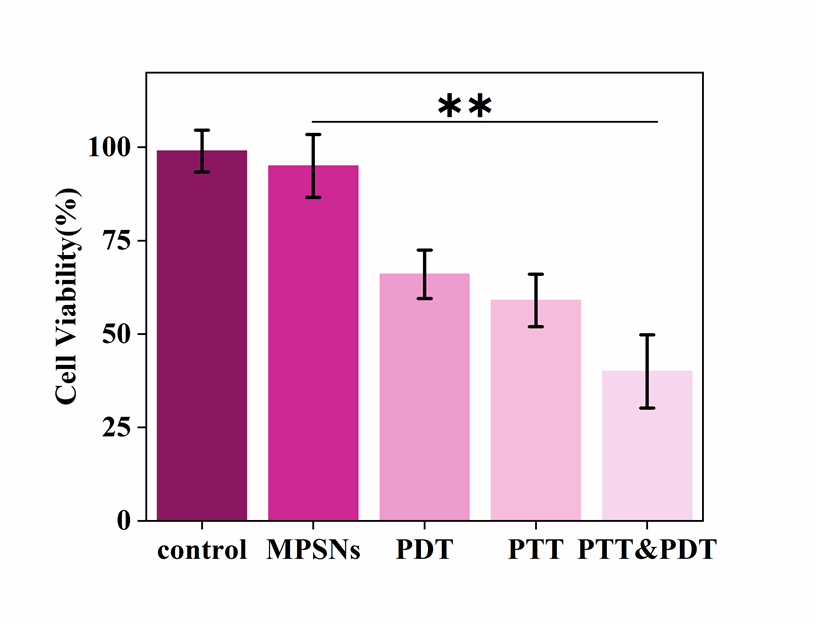
*

**Figure S7**. 4T1 cell viabilities after different treatments (PTT alone, PDT alone, and the combination of PTT and PDT) with MPSNs@R837 (R837=100 μg/mL) after 24 h of incubation (808 nm, 0.6 W/cm2). All data are mean ± SD (n = 3). Statistical significances were calculated via Student’s t test, **p < 0.01.


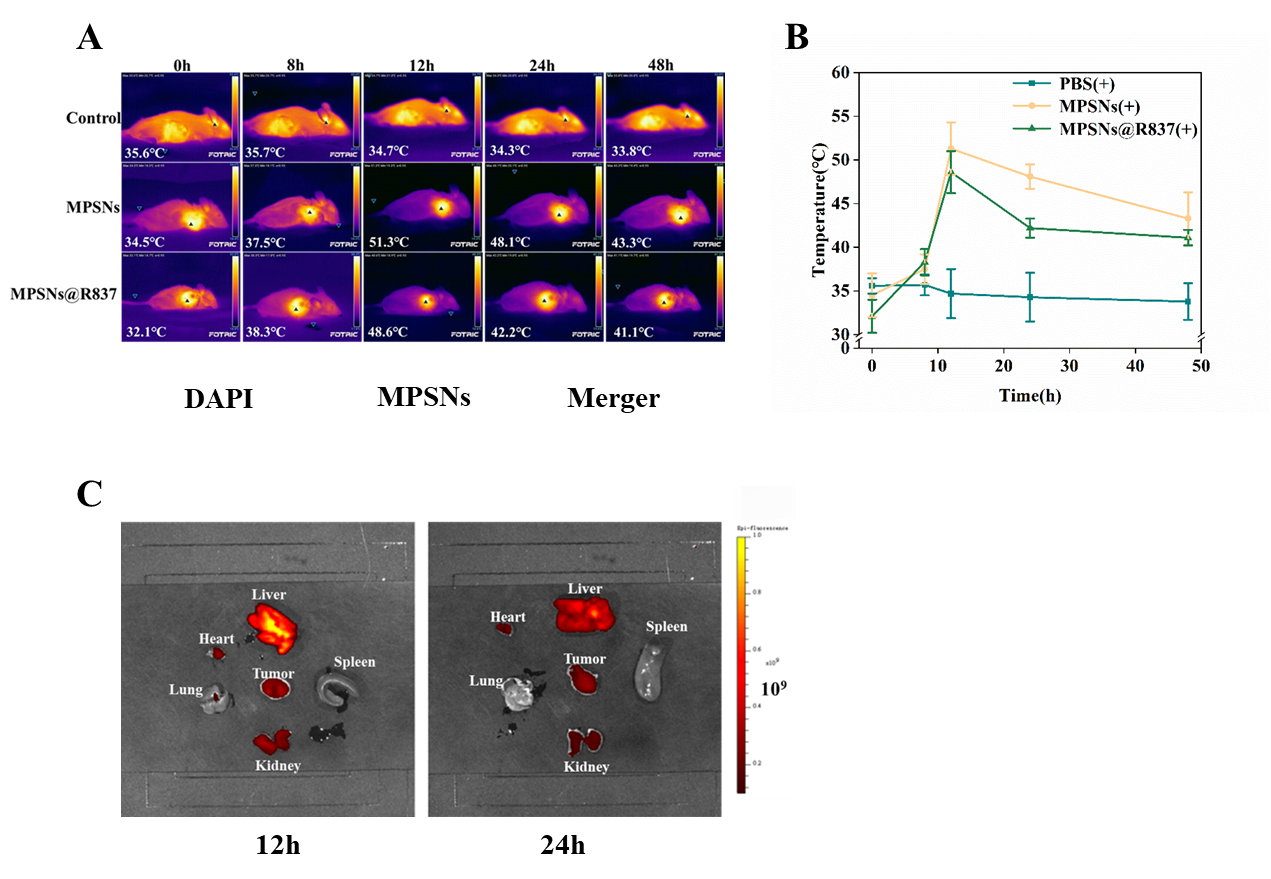


**Figure S8**. **A** Photothermal imaging after intravenous injection of saline, MPSNs and MPSNs@R837 in tumor-bearing mice. **B** Photothermal heating curves. **C** The ex vivo fluorescence images of tumors and major organs (heart, liver, spleen, lung and kidney) dissected from the mouse 12 and 24 h post injection. All data are mean ± SD (n = 5). Statistical significances were calculated via Student’s t test, ** p < 0.01.


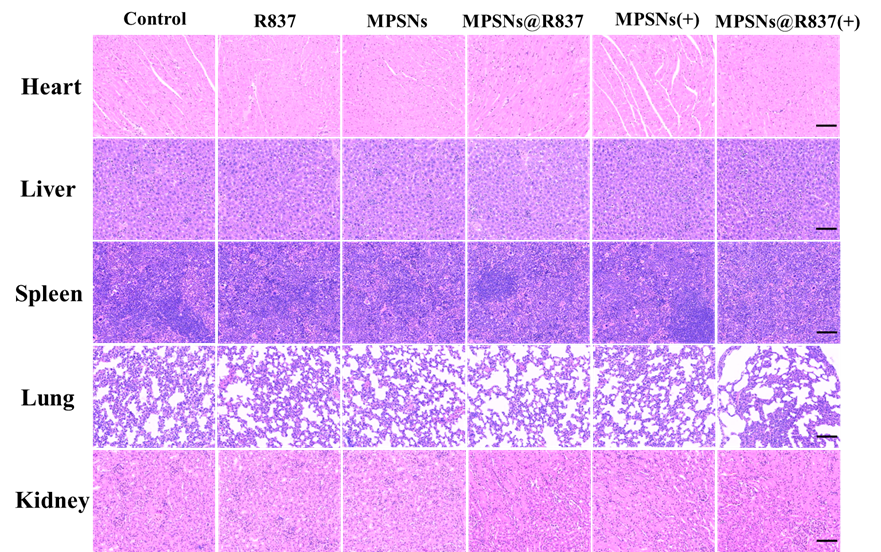


**Figure S9.** Representative H&E staining images of heart, liver, spleen, lung, and kidney of each treatment group at 21 days. (scale bar = 100μm)


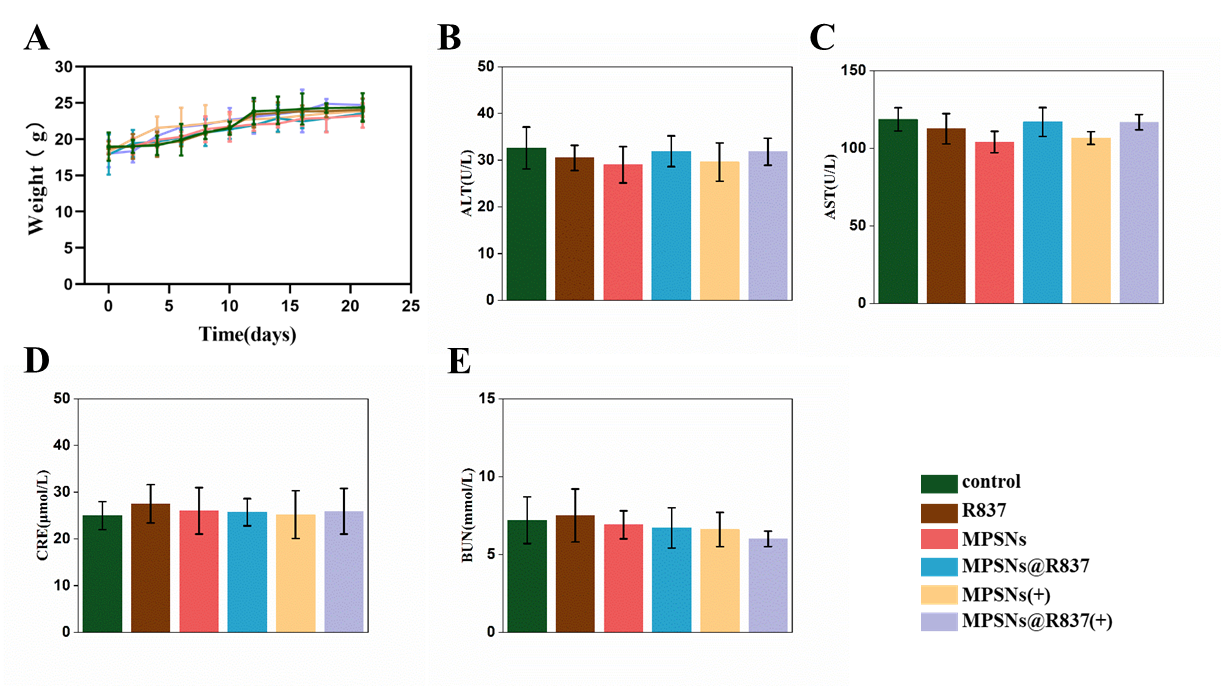


**Figure S10.** **A** Change of body weights of mice after different treatments. Serum biochemistry indicators: **B** alanine aminotransferase (ALT), **C** aspartate aminotransferase (AST), **D** creatinine (CRE), and (**E)** blood urea nitrogen (BUN) for each treatment group at 21.


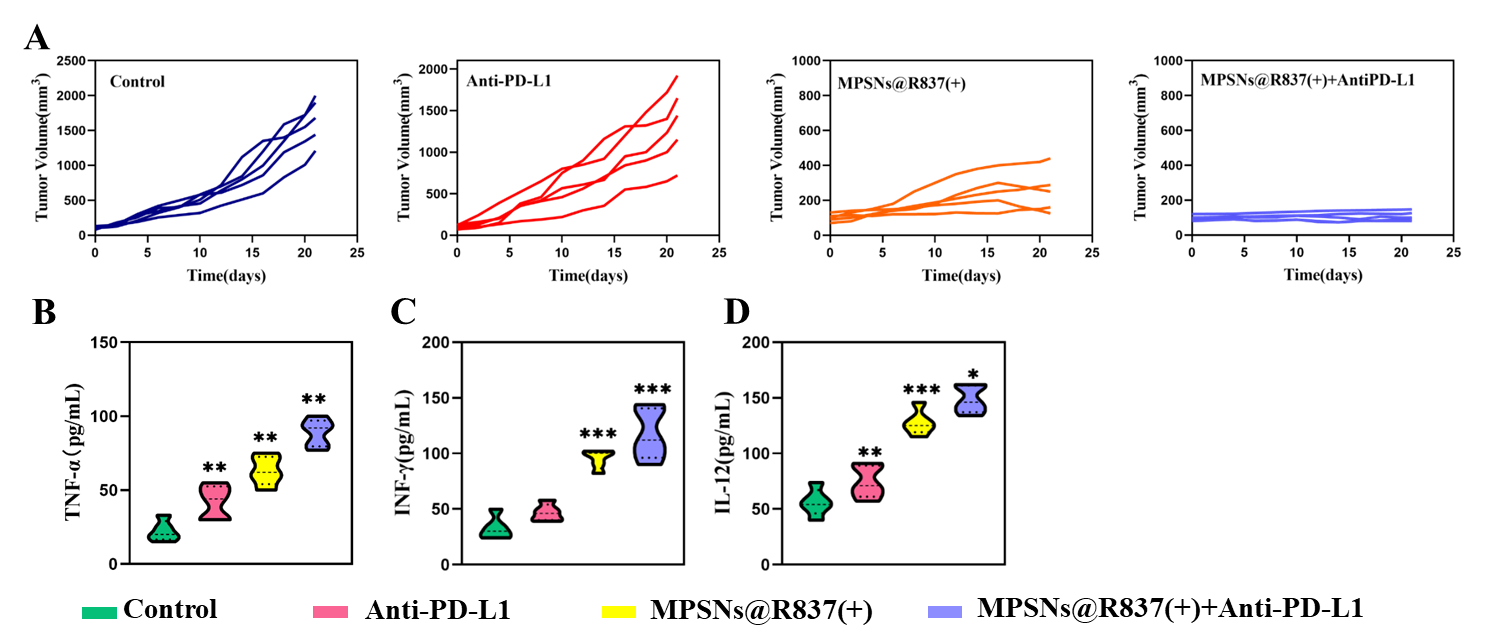


**Figure S11. A** Tumor volume. **B**-**D** cytokine levels of TNF-α, INF-γ and IL-12 in sera from mice.


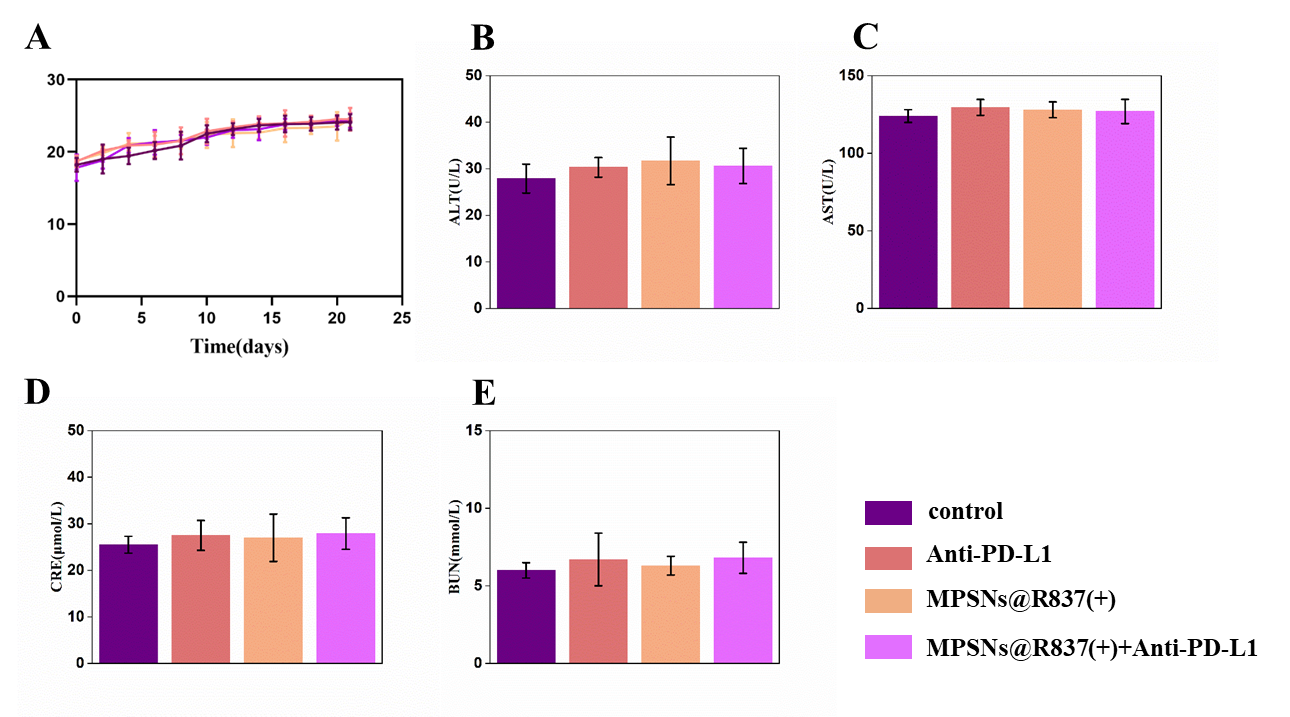


**Figure S12**. **A** Change of body weights of mice after different treatments. Serum biochemistry indicators: **B** alanine aminotransferase (ALT), **C** aspartate aminotransferase (AST), **D** creatinine (CRE), and (**E)** blood urea nitrogen (BUN) for each treatment group at 21 days.


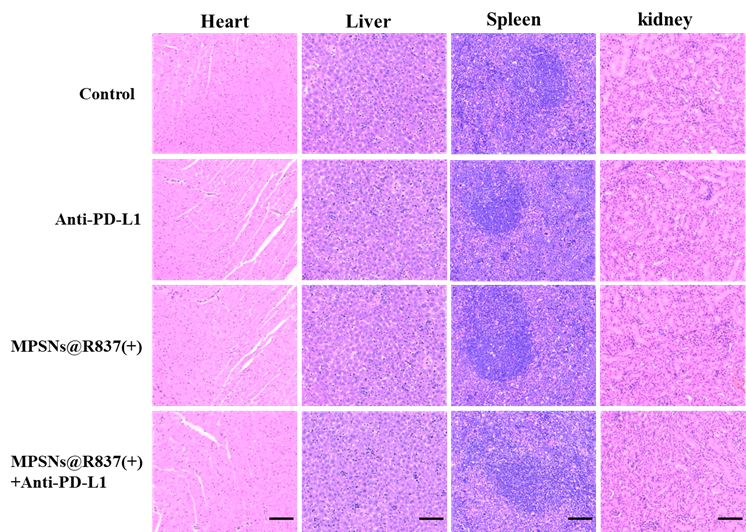


**Figure S13.** Representative H&E staining images of heart, liver, spleen, and kidney of each treatment group at 21 days. (scale bar = 100μm)


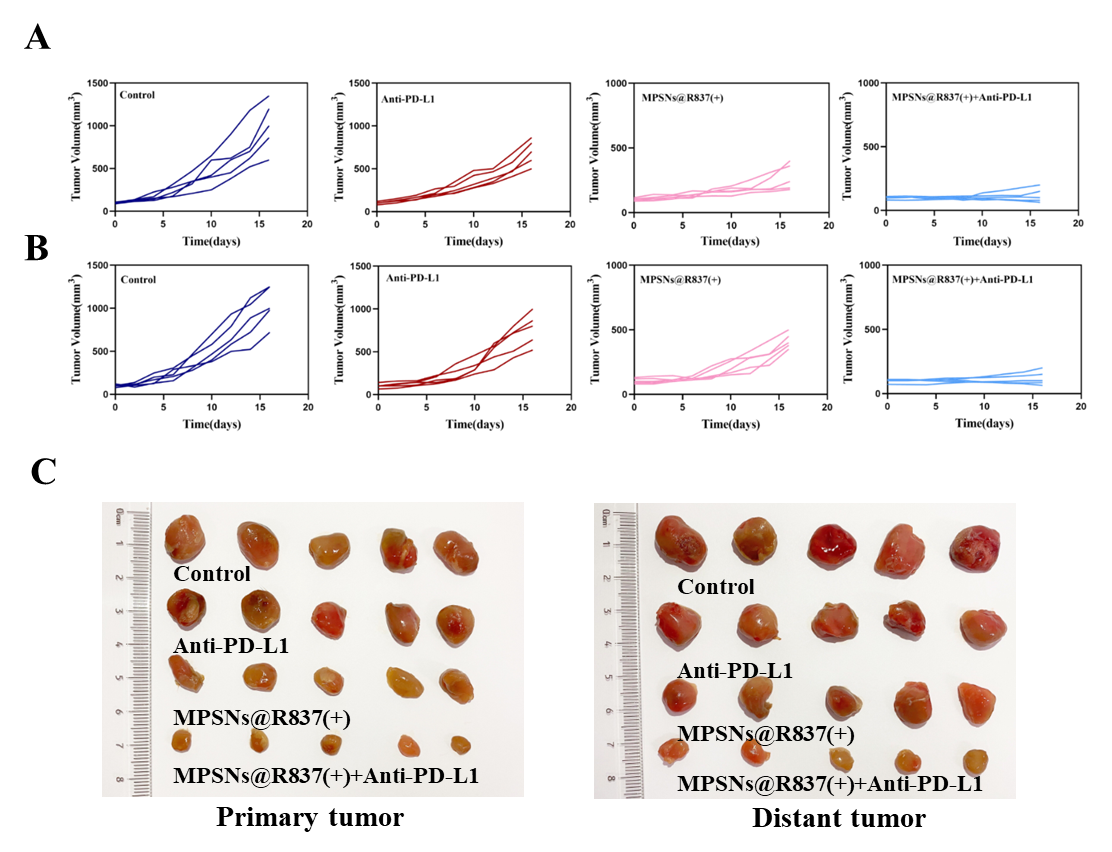


**Figure S14.** Tumor volume of **A** primary and (**B)** distant tumors of each group. **C** the images of tumors.


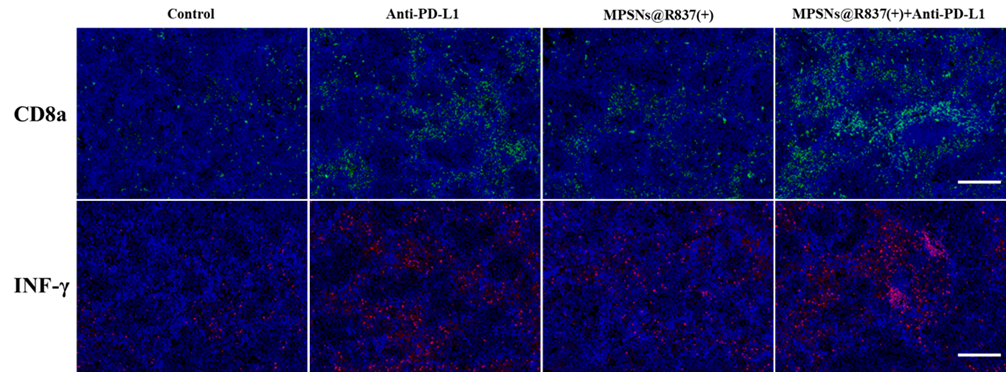


**Figure S15** Representative immunofluorescence staining for CD8a (green) and IFN-γ (red) of spleen sections (scale bar = 200μm).


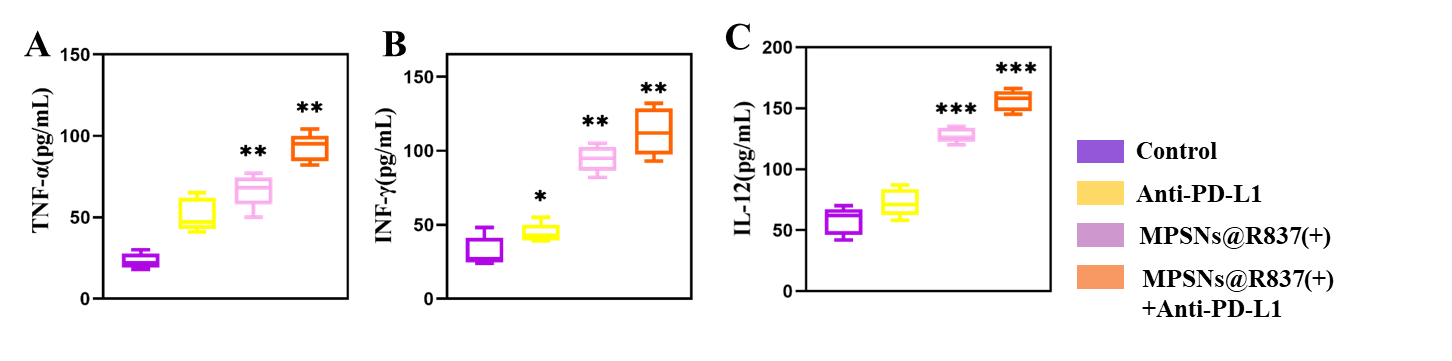


**Figure S16.** **A**-**C** cytokine levels of TNF-α, INF-γ and IL-12 in sera from mice.


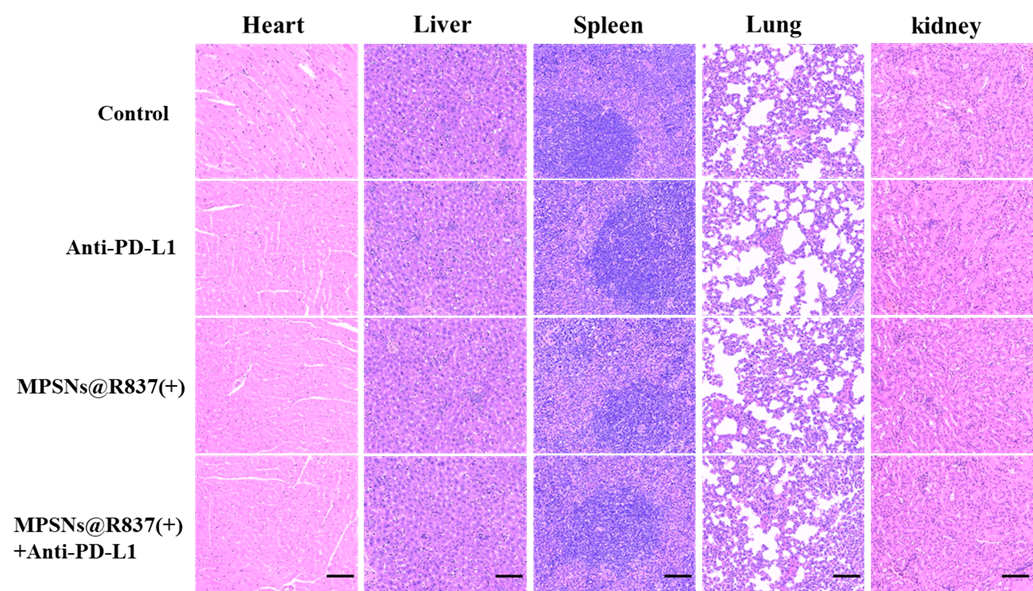


**Figure S17.** Representative H&E staining images of heart, liver, spleen, lung, and kidney of each treatment group at 16 days. (scale bar = 100μm)


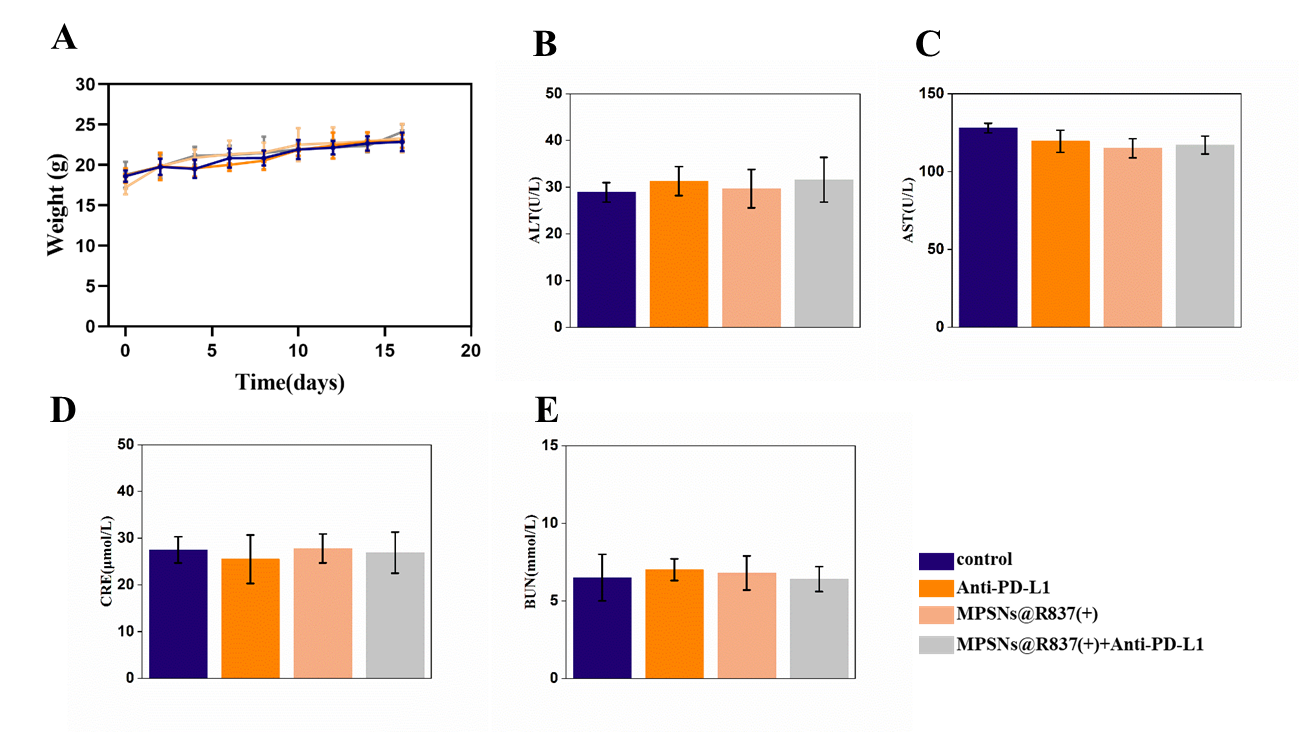


**Figure S18.** **A** Change of body weights of mice after different treatments. Serum biochemistry indicators: **B** alanine aminotransferase (ALT), **C** aspartate aminotransferase (AST), **D** creatinine (CRE), and (**E)** blood urea nitrogen (BUN) for each treatment group.
